# Treatment and outcome of chronic disseminated candidiasis in the era of antifungal prophylaxis: a retrospective multicentre cohort of 111 patients

**DOI:** 10.1016/j.eclinm.2026.104194

**Published:** 2026-09-16

**Authors:** Ilana Reinhold, Isabel Cristina Ramirez-Sanchez, Pao-Yu Chen, Yee-Chun Chen, Ankush Dhariwal, Jonathan Lambourne, Samir Agrawal, Maria Bastida, Carlota Gudiol, Serge Alfandari, Alessia Di Pilla, Sofya Khostelidi, Daniel Aguilar-Zapata, Aleksandra Barac, Ana Cipriano, Sabine Ehrlich, Jan Grothe, Jorge Illarramendi, Sara Jennrich, Reham Khedr, Dario Leotta, Gustavo-Adolfo Méndez, Julia A. Nacov, Miki Nagao, Guray Saydam, Stefan Schwartz, Jörg Steinmann, Kornelius Arn, David A. Enoch, Daniel Föhring, Oana Joean, Philipp Koehler, Robert Krause, Johan Maertens, Francesco Marchesi, Chizaram Onyeaghala, Neil Stone, Yuri Vanbiervliet, Nathan Wolfensberger, Luise Haensel, Julia Hurraß, Jens Panse, Ertan Sal, Jannik Stemler, Daniel Sabanés Bové, Rosanne Sprute, Maricela Valerio Minero, Antonio Vena, Livio Pagano, Oliver A. Cornely, Danila Seidel, FungiScope® Working Group of the European Confederation of Medical Mycology (ECMM) and the International Society for Human and Animal Mycology (ISHAM);, the Fungal Infections Study Group of the European Society of Clinical Microbiology and Infectious Diseases (EFISG) and the ESCMID Study Group for Infections in Compromised Hosts (ESGICH), Ilana Reinhold, Isabel Cristina Ramirez-Sanchez, Pao-Yu Chen, Yee-Chun Chen, Ankush Dhariwal, Jonathan Lambourne, Samir Agrawal, Maria Bastida, Carlota Gudiol, Serge Alfandari, Alessia Di Pilla, Sofya Khostelidi, Daniel Aguilar-Zapata, Aleksandra Barac, Ana Cipriano, Sabine Ehrlich, Jan Grothe, Jorge Illarramendi, Sara Jennrich, Reham Khedr, Dario Leotta, Gustavo-Adolfo Méndez, Julia A. Nacov, Miki Nagao, Guray Saydam, Stefan Schwartz, Jörg Steinmann, Kornelius Arn, David A. Enoch, Daniel Föhring, Oana Joean, Philipp Koehler, Robert Krause, Johan Maertens, Francesco Marchesi, Chizaram Onyeaghala, Neil Stone, Yuri Vanbiervliet, Nathan Wolfensberger, Luise Haensel, Julia Hurraß, Jens Panse, Ertan Sal, Jannik Stemler, Daniel Sabanés Bové, Rosanne Sprute, Maricela Valerio Minero, Antonio Vena, Livio Pagano, Oliver A. Cornely, Danila Seidel, Stefan Angermair, Denis Cetin, Natasa Colovic, Louise Cremer, Mara Dittrich-Salamon, Anna Doleschall, Ameni Elloumi, Uwe Klein, Emilie Mackevic, Alessandra Romano, Desiree Paul, Laman Rahimli, Jon Salmanton-García, Nadja Schnörch, Nur Akad Soyer, Reece Smith, Ming Tao Tsai, Natalia Vasenda, Alberto Vega Hernández, Chiara Wieser, Laura Zager

**Affiliations:** a Division of Infectious Diseases, Department I of Internal Medicine, Excellence Center for Medical Mycology (ECMM), Faculty of Medicine, University Hospital Cologne, Cologne, Germany; b Institute of Translational Research, Cologne Excellence Cluster on Cellular Stress Responses in Aging-Associated Diseases (CECAD), Faculty of Medicine, University of Cologne, Cologne, Germany; c Division of Infectious Diseases, Department of Internal Medicine, Universidad de Antioquia, Hospital Pablo Tobón Uribe, Medellín, Colombia; d Division of Infectious Diseases, Department of Internal Medicine, National Taiwan University Hospital, Taipei, Taiwan; e School of Medicine, National Taiwan Univeristy College of Medicine, Taipei, Taiwan; f National Institute of Infectious Diseases and Vaccinology, National Health Research Institutes, Miaoli, Taiwan; g Department of Infection, Barts Health NHS Trust, London, United Kingdom; h Department of Haemato-Oncology, St Bartholomew's Hospital, United Kingdom; i Blizard Institute, Queen Mary University of London, United Kingdom; j Department of Infectious Diseases, Bellvitge University Hospital, L'Hospitalet de Llobregat, Barcelona, Spain; k Bellvitge Biomedical Research Institute (IDIBELL), L'Hospitalet de Llobregat, Barcelona, Spain; l School of Medicine, University of Barcelona, Barcelona, Spain; m Centro de Investigación Biomédica en Red de Enfermedades Infecciosas (CIBERINFEC), Instituto de Salud Carlos III, Madrid, Spain; n Infectious Diseases Department, Hospital Center Gustave Dron, Tourcoing, France; o Department of Haematology, Agostino Gemelli University Hospital Foundation - IRCCS, Rome, Italy; p Catholic University of the Sacred Heart, Rome, Italy; q Department of Clinical Mycology, Allergology and Immunology, North-Western State Medical University named after I.I. Mechnikov, St. Petersburg, Russia; r Hospital Médica Sur, Infectious Diseases and Hospital Epidemiology, Mexico City, Mexico; s Clinic for Infectious and Tropical Diseases, University Clinical Center of Serbia, Belgrade, Serbia; t Department of Infectious Diseases, University Hospital Center de Santo António, Portugal; u Department of Medicine III, University Hospital, Ludwig Maximilian University (LMU) of Munich, Munich, Germany; v Department of Hematology and Cell Therapy, University Hospital Center of Bordeaux, Bordeaux, Nouvelle-Aquitaine, France; w Department of Hematology and Stem Cell Transplantation, West German Cancer Center Essen, University Hospital Essen, Essen, Germany; x Department of Pediatric Hematology/Oncology, National Cancer Institute- Cairo University, Egypt; y Department of Pediatric Hematology/Oncology, Bahrain Oncology Center - Kingdom of Bahrain; z Hematology Unit with BMT, A.O.U. University Hospital “G. Rodolico-San Marco”, Catania, Italy; aa Infectious Diseases Service, Teaching Hospital of Acute Care Dr. Ramón Madariaga, Posadas, Misiones, Argentina; ab Infectious Diseases Department, Faculty of Medicine, Catholic University of Misiones, Posadas, Misiones, Argentina; ac German Centre for Infection Research (DZIF), Partner Site Bonn-Cologne, Cologne, Germany; ad Department of Clinical Laboratory Medicine, Kyoto University Graduate School of Medicine, Kyoto, Japan; ae Department of Hematology, Ege University School of Medicine, Izmir, Turkey; af Department of Hematology, Oncology and Cancer Immunology, Charité - University Hospital Berlin, Corporate Member of Free University of Berlin and Humboldt University of Berlin, Berlin, Germany; ag Institute for Medical Microbiology, Infectious Diseases and Infection Control, Paracelsus Medical University, Nuremberg Hospital, Germany; ah Department of Hematology, Cantonal Hospital Luzern, Luzern, Switzerland; ai Clinical Microbiology & Public Health Laboratory, United Kingdom Health Security Agency (UKHSA), Cambridge University Hospitals NHS Foundation Trust, Cambridge, United Kingdom; aj Hematology, Kliniken Essen Mitte, Germany; ak Institute for Infectious Disease and Infection Control, Jena University Hospital, Friedrich-Schiller-University, Jena, Germany; al University of Cologne, Faculty of Medicine and University Hospital Cologne, Department I of Internal Medicine, Division of Clinical Immunology and Center for Integrated Oncology (CIO) Aachen Bonn Cologne Duesseldorf, Cologne, Germany; am Division of Infectious Disease, Department of Internal Medicine, Medical University of Graz, Graz, Austria; an Department of Microbiology, Immunology and Transplantation, KU Leuven, Leuven, Belgium; ao Department of Haematology, University Hospitals Leuven, Leuven, Belgium; ap Clinical and Experimental Oncohematology Unit, IRCCS Regina Elena National Cancer Institute, Rome, Italy; aq Department of Internal medicine, University of Port Harcourt Teaching Hospital, Port Harcourt, Rivers, Nigeria; ar Division of Infection, Hospital for Tropical Diseases, University College London Hospitals NHS Foundation Trust, London, United Kingdom; as Department I of Internal Medicine, Medical Faculty and University Hospital of Cologne University of Cologne, Cologne, Germany; at HYGIUM - Center for Hygiene and Environmental Medicine, Cologne, Germany; au Center for Integrated Oncology Aachen Bonn Cologne Duesseldorf (CIO ABCD) Aachen, Germany; av Department of Haematology, Oncology, Haemostaseology and Stem Cell Transplantation, University Hospital RWTH Aachen, Aachen, Germany; aw RCONIS/inferential.biostatistics GmbH, Friedrichstrasse 12, 4055 Basel, Switzerland; ax Clinical Microbiology and Infectious Diseases Department, Hospital General Universitario Gregorio Marañón, Madrid, Spain; ay Instituto de Investigación Sanitaria Gregorio Marañón, Madrid, Spain; az Facultad de Medicina, Universidad Complutense de Madrid, Madrid, Spain; ba Medicine Department, School of Medicine, Complutense University of Madrid, Madrid, Spain; bb Department of Health Sciences, University of Genoa, Genoa, Italy; bc Infectious Diseases Unit, IRCCS Hospital San Martino, Genoa, Italy; bd Faculty of Medicine and University Hospital Cologne, Clinical Trials Centre Cologne (ZKS Köln), University of Cologne, Cologne, Germany

**Keywords:** Hepatosplenic candidiasis, Haematological malignancy, Neutropenia, Candida, Antifungal therapy, Azole resistance

## Abstract

**Background:**

Chronic disseminated candidiasis (CDC) is a severe manifestation of invasive candidiasis predominantly affecting patients with haematological malignancies. Treatment recommendations are based on limited observational data, and the impact of widespread antifungal prophylaxis on CDC epidemiology and outcomes remains poorly characterised. We aimed to comprehensively characterise CDC and explore associations between antifungal treatment strategies and clinical outcomes.

**Methods:**

We performed a retrospective, multinational cohort study including adults with possible, probable, or proven CDC diagnosed between 2007 and 2025 identified through the FungiScope® registry (ClinicalTrials.gov: NCT01731353) and collaborating networks in 19 countries. Diagnostic findings, treatment strategies, and outcomes were analysed over 12 months. Exploratory comparative analyses assessed the association between antifungal treatment strategies and treatment response (composite of clinical, radiological, and mycological findings) and overall survival using inverse probability of treatment weighting to account for the time-varying confounders neutropenia and corticosteroid exposure.

**Findings:**

Of 111 patients, CDC was classified as possible in 23.4% (n = 26), probable in 50.5% (n = 56), and proven in 26.1% (n = 29) of patients. The predominant species were *Candida albicans* (32.4%, n = 36) and *Candida tropicalis* (24.3%, n = 27); no species was identified in 30.6% (n = 34). Breakthrough CDC occurred in 24.8% (27/109) of patients. PET/CT was used in 26.1% (n = 29) of patients for follow-up. Caspofungin (36.9%, 41/111) and liposomal amphotericin B (23/111; 20.7%) were the most frequently first-line agents for antifungal treatment. No statistically significant associations were detected between first-to-second-line treatment strategy, azole selection, and treatment response. Median treatment duration was 107 days (IQR 49–182), and no CDC relapse occurred after discontinuation. Corticosteroids for immune reconstitution inflammatory syndrome were used in 4.5% of patients. All-cause mortality was 33.3% (37/111), CDC-attributable mortality was 4.5% (5/111). Overall survival was higher among patients with breakthrough CDC than without (88.9% vs 58.5%; p = 0.004).

**Interpretation:**

No statistically significant associations between antifungal treatment strategy and treatment response or survival were detected in this retrospective dataset. However, the comparative treatment analyses were exploratory and the study was not powered to exclude clinically meaningful differences between antifungal strategies. Median treatment duration was shorter than previously reported. Future prospective studies integrating immunological biomarkers and the use of PET/CT-guided strategies are needed to optimise individualised treatment decision-making.

**Funding:**

This study received no dedicated external funding. FungiScope® receives unrestricted grants for registry maintenance and infrastructure from Basilea Pharmaceutica, Melinta Therapeutics LLC, Mundipharma Ltd., and Pfizer Inc, which had no role in the study design, conduct analysis, interpretation, manuscript preparation, or the decision to submit for publication.

**Research in context**

**Evidence before this study**

We searched PubMed for studies on chronic disseminated candidiasis (CDC) published from database inception to April 10, 2026 using the terms “chronic disseminated candidiasis”, “hepatosplenic candidiasis”, and “hepatic candidiasis”, without language restrictions. Published evidence in adult patients derives almost exclusively from single-centre retrospective cohorts and one small multicentre registry. A seminal Italian series of 28 patients published in 2002, before the introduction of mould-active prophylaxis, established CDC as a rarely fatal complication in which the principal morbidity was delay to haematological treatment rather than infection-attributable mortality. Subsequent cohorts from Taiwan, China, Israel, Italy and France, including up to 60 patients and all conducted within single countries, confirmed low CDC-attributable mortality but persistent uncertainty regarding optimal antifungal management. Radiological resolution rates ranged from 30 to 34% at 3 months to 48–57% at 6–9 months, supporting the convention of prolonged antifungal treatment for 3–6 months or until complete imaging resolution. The only prospective study, the multicentre French CANHPARI pilot study (n = 44), suggested that PET/CT normalisation at 3 months may better reflect resolution of active inflammation than conventional imaging, but was not designed to assess antifungal efficacy. No prospective randomised trial evaluating antifungal treatment strategies in CDC has been conducted. Current international guidelines recommend echinocandins or liposomal amphotericin B as initial therapy with oral azole step-down, but evidence supporting non-fluconazole azoles for long-term treatment, optimal treatment duration, and comparative antifungal efficacy after adjustment for time-varying confounders is absent. Moreover, the impact of widespread mould-active azole prophylaxis on CDC epidemiology, species distribution, and outcomes has not been evaluated in a large international cohort. In routine clinical practice, treatment courses are often prolonged and complex, driven by uncertainty in antifungal selection and by difficulty distinguishing persistent infection from immune reconstitution inflammatory syndrome on imaging. This uncertainty frequently results in extended therapy, with associated toxicity, cost, and delays to chemotherapy—the principal morbidity identified in earlier studies.

**Added value of this study**

This study represents, to our knowledge, the first multinational and largest CDC cohort including 111 adult patients from 19 countries, while accounting for time-varying confounders using marginal structural models. Previous single-centre cohorts identified shock, older age, and non-remission of the underlying malignancy as predictors of adverse outcomes, and suggested possible benefit from high-dose liposomal amphotericin B or caspofungin; however, none were adequately powered to account for time-varying confounding in treatment comparisons. In this retrospective cohort, exploratory analyses using marginal structural models with inverse probability of treatment weighting found no statistically significant associations between first-line antifungal class or oral azole step-down strategy (fluconazole versus non-fluconazole azoles) and treatment response or survival after accounting for the time-varying confounders neutropenia and corticosteroid exposure. Breakthrough CDC despite antifungal prophylaxis occurred in 24.8% of patients and was associated with improved survival, likely reflecting more favourable underlying haematological disease status. Median treatment duration was 107 days (IQR 49–182) and thus, substantially shorter than in previous cohorts, with no relapses observed after treatment discontinuation, suggesting that shorter treatment courses may be safe in selected patients. Finally, CDC-attributable mortality was low (4.5%), supporting previous observations that immune recovery rather than antifungal drug class is a major determinant of outcome, though larger prospective studies are needed to confirm this.

**Implications of all the available evidence**

Across cohorts spanning more than three decades, CDC-attributable mortality has consistently been low and outcomes were driven primarily by the underlying haematological disease and immune reconstitution rather than antifungal drug choice. Our findings extend this evidence in the largest international cohort from the era of mould-active antifungal prophylaxis with adjustment for clinically important confounders, although the comparative treatment analyses were exploratory.

These findings suggest greater flexibility in antifungal selection, particularly in patients with prior azole exposure or resistance. The absence of relapse following treatment discontinuation at a median of 107 days suggests that prolonged antifungal treatment until complete radiological resolution may not always be necessary in selected patients. Previous studies reported persistent CT abnormalities in up to 57% of patients at 9 months. Together with prior evidence, these findings further support the concept that conventional imaging is an unreliable marker of disease activity, whereas PET/CT may better reflect resolution of active inflammation. However, as the comparative treatment analyses were exploratory and not powered to exclude clinically meaningful differences between antifungal strategies, prospective studies are needed before optimal treatment strategies can be defined. Future prospective studies should incorporate immunological biomarkers of host immune reconstitution, including cytokine profiles and *Candida*-specific T-cell responses, together with PET/CT-guided assessment to enable individualised management strategies, including antifungal therapy and identification of patients who may benefit from adjunctive immunomodulatory therapies in patients with CDC.

## Introduction

Chronic disseminated candidiasis (CDC), also referred to as hepatosplenic candidiasis, is a distinct manifestation of invasive candidiasis occurring predominantly in patients with haematological malignancies during recovery from prolonged neutropenia.^1^ The epidemiology of CDC is evolving in the context of widespread mould-active triazole prophylaxis, with a declining incidence in acute myeloid leukaemia (AML) and growing proportion of cases in patients with lymphoma and acute lymphoblastic leukaemia (ALL).^2^, ^3^, ^4^, ^5^ Established risk factors include intensive induction chemotherapy, severe and prolonged neutropenia, broad-spectrum antibiotic exposure, and central venous catheter use.^2^^,^^4^, ^5^, ^6^

CDC typically presents with persistent fever, abdominal pain, cholestasis, and characteristic hepatosplenic abscesses on imaging.^3^^,^^6^^,^^7^ Microbiological confirmation is obtained in only a minority of patients, and most patients meet criteria for possible rather than proven or probable disease according to the EORTC/MSGERC consensus definitions.^2^, ^3^, ^4^^,^^7^^,^^8^ Diagnostic sensitivity may be improved by imaging modalities beyond ultrasound, including computed tomography (CT), magnetic resonance imaging (MRI), and fluorodeoxyglucose positron emission tomography-CT (FDG-PET/CT), and non-culture biomarkers such as β-D-glucan (BDG) and mannan antigen/antibody testing, but their optimal integration into diagnostic algorithms remains incompletely defined.^9^, ^10^, ^11^

Current international guidelines recommend intravenous echinocandins or liposomal amphotericin B as initial therapy for CDC and subsequent switch to oral treatment with azoles after several weeks and control of symptoms.^12^^,^^13^ The administration of azole other than fluconazole is supported by limited evidence which is mainly derived from small observational studies.^12^, ^13^, ^14^ Prior azole exposure should be carefully considered when selecting subsequent treatment, as it may alter *Candida* epidemiology and reduce susceptibility, potentially leading to treatment failure.^15^^,^^16^ This is particularly relevant in haematological patients, in whom mould-active azole prophylaxis is broadly used and azole-resistance is increasing.^17^

Treatment is typically prolonged and guided by serial imaging until lesion resolution; however available data is heterogeneous with median treatment durations ranging from 4 to 7 months.^2^^,^^4^^,^^12^^,^^18^ Radiological resolution of lesions often lags behind clinical improvement and may take several months.^13^ Although CDC diagnosis can lead to modification or delay of chemotherapy, this is not recommended by guidelines as it may have potential adverse consequences for underlying disease outcomes.^4^^,^^5^^,^^12^

Published data on CDC are predominantly country-specific retrospective studies.^3^^,^^4^^,^^6^^,^^7^^,^^19^ Comparative evidence regarding antifungal treatment strategies, optimal treatment duration, and response assessment remains limited. To address these knowledge gaps and inform contemporary clinical management of CDC in the era of mould-active antifungal prophylaxis and increasing azole resistance, we conducted a multinational retrospective cohort study of adults with CDC. The primary objective was to evaluate antifungal treatment strategies, including long-term options beyond fluconazole, and their association with treatment response and overall survival. Additional objectives were to describe *Candida* species distribution and antifungal resistance, assess the contribution of advanced imaging modalities, characterise breakthrough CDC, and identify prognostic factors for treatment failure and death.

## Methods

### Study population and definitions

Patients aged ≥18 years who received antifungal therapy for possible, probable, or proven CDC between January 2007 and December 2025 were eligible. CDC was classified according to the 2020 EORTC/MSGERC consensus definitions.^8^ Proven CDC required a host factor (haematological malignancy and/or neutropenia) and histopathological or microbiological (culture, direct microscopy or PCR) confirmation of *Candida* spp. from liver biopsy specimens; probable CDC required a host factor, compatible imaging in spleen or liver plus positive serum BDG or candidaemia within the preceding 14 days; possible CDC required a host factor and compatible imaging findings. Breakthrough CDC was defined as disease occurring despite systemic antifungal prophylaxis administered for ≥5 days within 14 days preceding diagnosis. Day 0 was defined as the date of initiation of targeted antifungal therapy for CDC. Antifungal therapy administered prior to day 0 was defined as empirical treatment. Combined therapy was defined as an overlap for >3 days between two antifungal agents. For outcome evaluation, only first-line antifungal drugs for >3 days were included in treatment response and mortality analysis.

### Ethics

FungiScope® (ClinicalTrials.gov: NCT01731353) is a global web-based registry for invasive fungal infections approved by the Ethics Committee of the University Hospital Cologne, Germany (Study ID: 05-102). As the study is retrospective, informed consent was waived.

### Patient identification and data collection

Patients were identified through an outreach via scientific society networks (Supplementary Appendix), personal investigator invitations, and the FungiScope® network. Collaborating investigators were asked to propose all patients at their site potentially meeting the CDC case definition. Details on case identification, validation, and duplicate-case review are provided in the Supplementary Methods. Data were collected retrospectively using a study-specific electronic case report form (for variables, see Supplementary).

Treatment response, neutropenia, and immunosuppression were assessed on days 14, 28, 42, and 84, and at 6, 9, and 12 months. Treatment response was recorded as a single, investigator-assigned category (complete response, partial response, stable disease, or progressive disease) on the electronic case report form (Supplementary Table S1), reflecting the treating investigator's overall judgement informed by available clinical, radiological, and mycological findings. Clinical, radiological, and mycological response were not captured as separate, independently adjudicated domains. Treatment failure was defined as stable or progressive disease, whereas treatment success comprised partial and complete responses in accordance with the EORTC-MSGERC criteria.^20^ Relapse following discontinuation of antifungal therapy was recorded. All-cause and CDC-attributable mortality were documented, as adjudicated by investigators. No central blinded adjudication was undertaken.

### Statistical analysis

Statistical analyses were performed using SPSS Statistics version 30 (IBM, USA) and R programming language (v 4.5.2). Continuous variables were summarised as medians with interquartile ranges and categorical variables as frequencies and percentages. Continuous variables were compared using the Mann–Whitney U test. Categorical variables were analysed using the Chi-squared test or Fisher's exact test, where appropriate. Overall survival was estimated using the Kaplan–Meier method, comparing patient groups defined by haematological/oncological disease status (de novo vs. other) at diagnosis, first-line antifungal treatment class, and prolonged (≥10 days) neutropenia within 30 days prior to diagnosis. To compare results with a previously published analysis,^2^ a Cox proportional hazards model was fitted including baseline antifungal treatment (azole vs. echinocandin), adjunctive corticosteroid use, age at diagnosis, neutropenia within 30 days prior to diagnosis, and haematological disease status at diagnosis. Hazard ratios (HRs) with 95% confidence intervals (CI) were reported. Patients were censored at last follow-up if alive.

To evaluate treatment response over time, marginal structural modelling with stabilised inverse probability of treatment weights (IPTW) was used to address time-varying confounding by neutropenia and corticosteroid exposure; response was assessed at day 84, month 6, month 9, and month 12. Baseline covariates were explored individually in the weighted model, with p-values adjusted using the Benjamini-Hochberg procedure. One antifungal class was assigned to each assessment interval according to the agent administered for the longest duration during that interval defining the dominant antifungal class; shorter overlapping treatments were not modelled separately. Separate propensity score-weighted models were estimated among patients undergoing a first to-second line treatment switch, to compare three treatment strategies analysing transitions from echinocandins or amphotericin B to azoles, with treatment response at day 84 as the primary outcome. Patients therefore contributed observations to successive intervals only if their treatment changed over time. As sensitivity analyses, all statistical analyses were repeated after restricting the cohort to (i) patients with proven or probable CDC, excluding those classified as possible CDC; (ii) patients with a known *Candida* species, excluding those with unknown species; and (iii) patients without combination therapy (i.e., overlapping antifungal treatment) during the treatment course. Full methodological details are provided in the Supplementary Appendix.

### Role of funding source

This study received no dedicated external funding. FungiScope® receives unrestricted grants for registry maintenance and infrastructure from Basilea Pharmaceutica, Melinta Therapeutics LLC, Mundipharma Ltd., and Pfizer Inc, which had no role in the study design, conduct analysis, interpretation, manuscript preparation, or the decision to submit for publication.

## Results

Between July 31, 2025 and January 10, 2026, investigators from participating centres retrospectively proposed patients meeting the CDC case definition. All submitted cases underwent central review, and cases not fulfilling case definition were excluded (see Supplementary). The final cohort comprised 111 eligible patients from 19 countries (Supplementary Table S2), the majority from Germany (25/111, 22.5%), Taiwan (13/111, 11.7%), Colombia (11/111, 9.9%), France (10/111, 9.0%), and the United Kingdom (10/111; 9.0%). Most patients were diagnosed between 2023 and 2025 (43/111, 38.8%; Supplementary Figure S1).

### Patient characteristics

Baseline characteristics of the 111 patients are shown in Table 1. The most frequent underlying haematological malignancies were AML (53/109, 48.6%), ALL (27/109, 24.7%), and lymphoma (17/109, 15.6%); two patients had no underlying haematological malignancy and presented with severe neutropenia induced by metamizole. Breakthrough CDC occurred in 27/109 patients (24.8%). There was no significant difference in the occurrence of breakthrough CDC between AML and non-AML patients (18.9% vs. 29.3%; OR 1.78, p = 0.27) (Supplementary Table S3).

**Table 1:** Baseline characteristics of 111 patients with chronic disseminated candidiasis.

Characteristic	N	%
Age at diagnosis (years), median (IQR)	49 (36–59)	
Male	57	51.4
Female	54	48.6
Underlying disease		
Haematological/oncological disease	109	98.2
Acute myeloid leukaemia^a^	53	48.6
Acute lymphoblastic leukaemia	27	24.7
Lymphoma^b^	17	15.6
Multiple myeloma	2	1.8
Myelodysplastic syndrome	5	4.6
Chronic lymphocytic leukaemia	1	0.9
Chronic myelogenous leukaemia	1	0.9
Aplastic anaemia	1	0.9
Solid tumour	2	1.8
Months of haematological/oncological diagnosis prior CDC, median (IQR)	2 (1–8)	
Haematological/oncological disease state, n = 105		
De novo (first line)	53	48.6
First relapse after complete remission	25	22.9
First progression	4	3.7
Second or later relapse/progression	17	15.6
Primary refractory	6	5.5
Autologous haematopoietic cell transplantation	9	8.3
Days since HCT, median (IQR)	45 (23–404)	
Allogeneic haematopoietic cell transplantation	16	14.7
Days since HCT, median (IQR)	34 (10.5–394.5)	
Acute GvHD within 90 days^c^	3	2.8
Chronic GvHD within 90 days^c^	2	1.9
Mucositis grade III/IV within 30 days	34	31.2
HIV, unsuppressed	1	0.9
Neutropenia (<500 cells/μl) at CDC diagnosis	53	47.7
Prolonged neutropenia <500 cells/μl (≥10 days within prior 30 days)	92	82.9
Uncontrolled diabetes mellitus	1	0.9
ICU stay at diagnosis of CDC	20	18.0
Medication prior diagnosis of CDC		
Corticosteroid ≥0.3 mg/kg/day prednisone equivalent for ≥3 weeks (within 60 days)	34	30.6
Antibiotics in previous 14 days	73	65.8
Other immunosuppressive drugs (within 3 months)^d^	13	11.7
Antifungal prophylaxis prior CDC diagnosis^e^	27	24.8
Fluconazole	14	12.8
Voriconazole	6	5.5
Caspofungin	3	2.8
Posaconazole	2	1.8
Amphotericin B liposomal	1	0.9
Amphotericin B lipid complex	1	0.9

Percentages in Table are calculated based on available data, excluding missing values.

CDC: chronic disseminated candidiasis; GvHD: graft versus host disease; HCT: haematopoietic cell transplantation; ICU: intensive care unit.

a Among patients with AML and information on disease stage, 23/52 (44.2%) had de novo disease, 14/52 (26.9%) were in first relapse after complete remission, 8/52 (15.4%) had ≥2nd relapse/progression, 5/52 (9.6%) had primary refractory disease, and 2/52 (3.8%) had first progression. Median time since AML diagnosis to CDC diagnosis was 3 months (IQR: 1–6).

b Among patients with lymphoma (n = 17) subtype information was available in 16/17: diffuse large B-cell lymphoma was most common (5/17, 29.4%), followed by other B-cell lymphoma (4/17, 23.5%), T-cell lymphoma (4/17, 23.5%), Hodgkin lymphoma (3/17, 17.6%).

c GvHD, within 90 days prior to CDC diagnosis: grade III colon (n = 3) and grade III liver (n = 2).

d Non-chemotherapy immunosuppressive drugs included anti-thymocyte globuline, cyclosporine, tacrolimus, daratumumab, inotuzumab-ozogamicin, and rituximab.

e Antifungal drugs administered as prophylaxis within 14 days prior CDC diagnosis for ≥5 days. Information on antifungal prophylaxis was missing in 2 cases. Breakthrough cases occurred in patients with AML (10/27, 37.0%), ALL (5/27, 18.5%), lymphoma (6/27, 22.2%), CML (1/27, 3.7%), multiple myeloma (1/27, 3.7%), MDS (3/27, 11.1%), and solid tumour (1/27, 3.7%).

CDC classification, affected sites and diagnostics are summarised in Table 2. BDG was measured at baseline in 35 patients (31.5%), of whom 31 (88.6%) had positive results. Among the 22 patients in whom BDG was reassessed after at least 28 days after CDC diagnosis, values remained persistently positive in 16 (72.7%) patients and became negative in six (27.3%).

**Table 2:** Clinical, mycological and imaging characteristics of 111 patients with chronic disseminated candidiasis.

Characteristics	Category	Patients (N)	%	Total examinations (N)
CDC classification	Proven	29	26.1	
	Probable	56	50.5	
	Possible	26	23.4	
*Candida* species	*C. albicans*	36	32.4	
	*C. glabrata (Nakaseomyces glabratus)*	1	0.9	
	*C. krusei (Pichia kudriavzevii)*	2	1.8	
	*C. parapsilosis*	5	4.5	
	*C. tropicalis*	27	24.3	
	*C. dubliniensis*	5	4.5	
	*C. lusitaniae (Clavispora lusitaniae)*	1	0.9	
	Unknown (culture/PCR negative)	34	30.6	
Affected sites attributed to CDC^a^	Liver	102	91.9	
	Spleen	83	74.8	
	Lung	23	20.7	
	Kidneys	21	18.9	
	Central nervous system	9	8.1	
	Skin and soft tissue^b^	9	8.1	
	Eye^c^	2	1.8	
	Heart valves	1	0.9	
	Intestines	1	0.9	
	Peritoneum	1	0.9	
Imaging modalities throughout the follow-up	CT	93	83.8	293
	MRI	31	27.9	62
	PET/CT	29	26.1	56
	Ultrasound (US)	45	40.5	72
	Contrast-enhanced US	6	5.4	8
	Ophthalmoscopy	4	3.6	4
Mycological diagnostics	Blood culture positive for *Candida* spp. within 14 days prior CDC	33	29.7	
	Liver biopsy^d^	38	34.2	69
	Transjugular	7	6.3	15
	Percutaneous	32	28.8	54
	Positive result	29	26.1	40
	Antifungal susceptibility testing available	52	46.8	
	Fluconazole resistance	11/52	21.2	
	Echinocandin resistance	1/52	1.9	
Beta-D-Glucan	BDG measurement at baseline	35	31.5	
	BDG positive at baseline	31/35	88.6	
	BDG persistently positive ≥28 d^e^	16/22	72.7	
	BDG negative after ≥28 d	6/22	27.3	
**Liver parameters at CDC diagnosis**	**Category**	**Median values (IQR)**	**Abnormal values n/N (%)**^f^	

	AST (U/L), median (IQR)	25 (20–40.5)	22/84 (26.2)	
	ALT (U/L), median (IQR)	34 (19–63)	42/97 (43.3)	
	ALP (U/L), median (IQR)	166.5 (36–295)	51/92 (55.4)	
	GGT (U/L), median (IQR)	115 (58–285)	71/85 (83.5)	
	Bilirubin, total (mg/dL), median (IQR)	1.1 (0.64–2.64)	42/93 (45.2)	
	Albumin (g/dL), median (IQR)	3 (2.5–4.35)	76/80 (95)	

AST: aspartate aminotransferase; ALT: alanine aminotransferase; ALP: alkaline phosphatase; BDG: Beta-D-Glucan; CDC: chronic disseminated candidiasis; GGT: gamma-glutamyl transferase. Abnormal values were defined according to the following reference ranges: AST <40 U/L, ALT <40 U/L, ALP <147 U/L, GGT <50 U/L, bilirubin, total <1.2 mg/dL, albumin >3.5 g/dL.

a Mandatory site involvement for study eligibility: liver or spleen.

b Skin biopsy was performed in n = 5 patients (4.5%), with yeast confirmed by histopathology, culture and/or PCR in 4/5 samples.

c Ophthalmoscopy was performed in n = 4 (3.6%) patients. Ocular involvement attributable to CDC was identified in two of these patients; the remaining two examinations showed no evidence of ocular candidiasis.

d Fifteen patients underwent more than one liver biopsy procedure: transjugular in n = 3, percutaneous in n = 11, and both transjugular and percutaneous in n = 1. Positive results from liver biopsy in n = 29 were obtained by histopathology (evidence of yeast) in n = 20 patients, culture in n = 9, diagnostic PCR in n = 9, and direct microscopy in n = 3.

e BDG measurement was not repeated after ≥28 d in n = 13.

f Denominators vary due to missing data in testing of laboratory parameters.

**Fig. 1:**
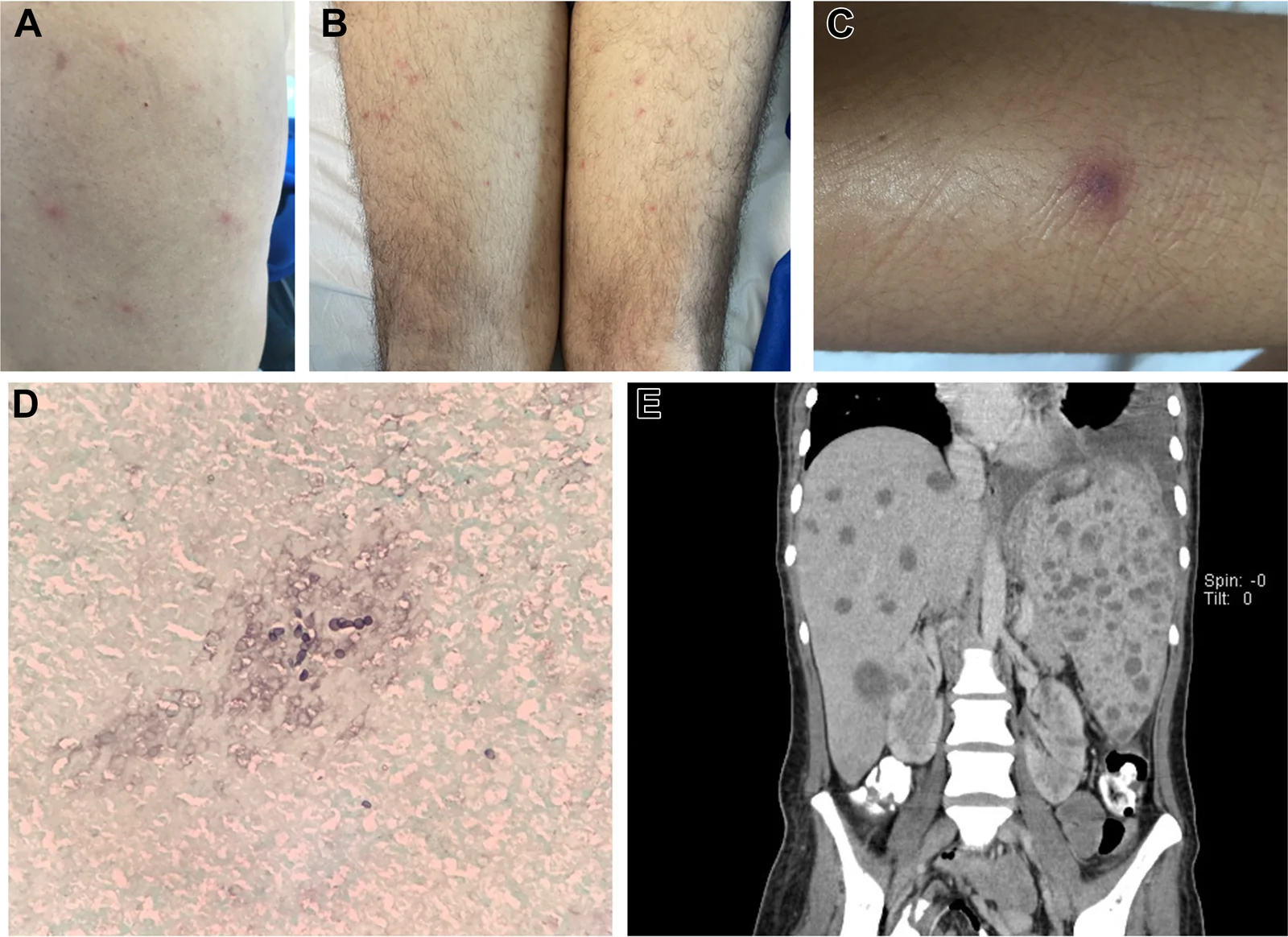
**Disseminated *Candida tropicalis* infection in a patient with cutaneous, hepatosplenic, and renal involvement.** (A–C): Erythematous papular-to-nodular skin lesions on the back (A), legs (B), and arm (C). (D): Liver biopsy showing numerous budding yeast forms consistent with *Candida* species (Grocott-Gomori methenamine silver stain). (E): Abdominal computed tomography demonstrating multiple hypodense lesions in the liver, spleen, and kidneys, consistent with disseminated candidiasis.

Nine patients showed central nervous system (CNS) involvement beyond hepatic or splenic disease. Neuroimaging demonstrated multifocal punctate or ring-enhancing lesions consistent with septic embolic dissemination or diffuse pachymeningeal thickening. In some patients, non-enhancing lesions suggestive of embolic infarctions were observed. Cerebrospinal fluid BDG testing was positive in two of the patients undergoing lumbar puncture. Among the four patients who underwent ophthalmoscopy, ocular involvement attributable to CDC was identified in two. Skin or soft tissue involvement was observed in nine patients, including six with *Candida tropicalis* infection. Clinical findings are shown in Fig. 1.

### Species distribution and antifungal resistance

*Candida* species distribution is presented in Table 2. Notably, 27 patients (24.3%) were attributed to *C. tropicalis*, predominantly from Taiwan (9/27, 33.3%) and Colombia (8/27, 29.6%).

Fluconazole resistance was classified according to the local laboratory interpretation; details of antifungal susceptibility testing methods are provided in the Supplementary. Fluconazole resistance was identified in 11 *Candida* isolates and was more frequent in *C. tropicalis* than in other species (8/27 [29.6%] vs. 3/84 [3.6%]; p < 0.001). Fluconazole-resistance was also detected in *Candida parapsilosis* (1/5), *Candida glabrata* (1/1), and *Candida albicans* (1/36, 2.8%). One echinocandin-resistant *C. albicans* isolate harbouring an *FKS* mutation was identified. Among patients with fluconazole resistance, 7/11 (63.6%) were associated with breakthrough CDC.

### Imaging

Within the first week after CDC diagnosis, CT was the predominant imaging modality (n = 96), followed by ultrasound (n = 30), MRI (n = 14), and PET/CT (n = 13), while contrast-enhanced ultrasound (n = 3) was rarely used. Baseline findings most commonly included hypodense hepatic or splenic lesions, followed by nodular and microabscess patterns.

The first follow-up imaging was performed after a median of 14 days (IQR 7–24). Among 74 patients with evaluable imaging, progression occurred in 28 (37.8%), stable disease in 26 (35.1%), improvement in 17 (23.0%), and complete response in 3 (4.1%). Over the disease course, patients underwent a median of four imaging procedures (IQR 3–6), with a median follow-up of 85 days (IQR 39–189, maximum 4.4 years). At last imaging (n = 97), complete response was observed in 30 patients (30.9%), improvement in 27 (27.8%), stable disease in 21 (21.6%), and progression in 19 patients (19.6%).

PET/CT was performed in 29 patients (26.1%) during follow-up. Patients who underwent PET/CT had longer overall antifungal treatment durations than those who did not (n = 82) (median 138 vs. 88 days, p = 0.02). In 12 of these 29 patients (41.4%), regression of PET/CT findings was explicitly cited as the reason for antifungal treatment discontinuation. These patients had longer treatment durations than patients without PET-guided treatment modifications (median 219 vs. 88 days, p = 0.001).

### Antifungal treatment

**Fig. 2:**
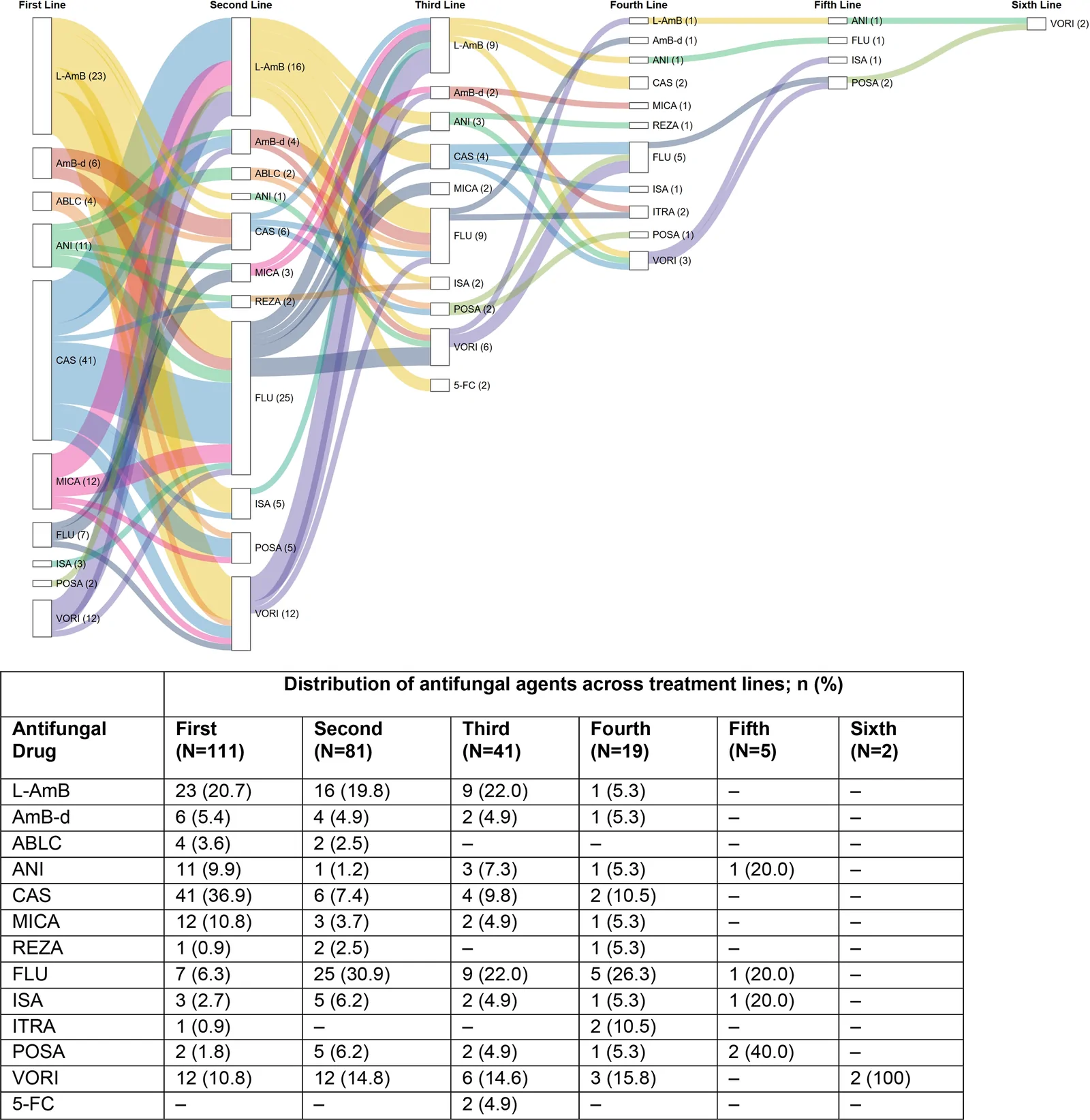
**Antifungal treatment lines in 111 patients with chronic disseminated candidiasis.** Sankey diagram illustrating antifungal use from first- to sixth-line therapy. Each column represents a treatment line, with rectangles indicating antifungal agents. Flow widths are proportional to the number of patients transitioning between therapies. The table summarises the distribution of antifungal agents per treatment line. No treatment transition was observed in 30 patients, with caspofungin discontinued without transition in 14/111 (12.6%) patients. Reasons for first-line therapy modifications were switch to oral administration (16/81, 19.8%), worsening of clinical condition (15/81, 18.5%), worsening in imaging (10/81, 12.3%), worsened inflammatory parameters (10/81, 12.3%), improvement in imaging (7/81, 8.6%), improvement of clinical condition (7/81, 8.6%), availability of susceptibility test results (7/81, 8.6%), improved inflammatory parameters (5/81, 6.2%), drug-related adverse events (4/81, 4.9%), and haemodynamic stability (2/81, 2.5%). No treatment modifications were attributed to a predefined duration of initial antifungal therapy, haemodynamic instability, or switch to outpatient parenteral antimicrobial therapy. Multiple reasons per case were possible. Combination antifungal therapy, defined as an overlap of more than 3 days between two antifungal agents within the same patient, occurred in 22 patients, accounting for 43 overlapping treatment episodes. These therapies are not shown in the Sankey diagram but are detailed in Supplementary Table S4. Abbreviations: AmB-d: Amphotericin B deoxycholate; ABLC: Amphotericin B lipid complex; L-AmB: Amphotericin B liposomal; ANI: Anidulafungin; CAS: Caspofungin; MICA: Micafungin; REZA: Rezafungin; FLU: Fluconazole; ISA: Isavuconazole; ITRA: Itraconazole; POSA: Posaconazole; VORI: Voriconazole; 5-FC: Flucytosine.

As first-line antifungal treatment, caspofungin was the most frequent choice (41/111, 36.9%), followed by liposomal amphotericin B (23/111, 20.7%) and micafungin (12/111, 10.8%). Antifungal treatment course, including modifications of treatment lines, is shown in Fig. 2. Transition from first to second treatment line occurred in 81 (73.0%) patients. The median duration of first line antifungal therapy prior to modification was 18.5 days (IQR 8.8–42.0; mean 43.7 days). The most frequent first-line modifications were transitions from echinocandins to azoles (23/81, 28.4%), including fluconazole (15/81, 18.5%) and other azoles (8/81, 9.9%). Transitions from polyenes to azoles occurred in 13/81 patients (16.0%) to fluconazole and 8/81 (9.9%) to other azoles. Switching from echinocandins to polyenes was observed in 16/81 patients (19.8%).

Combination antifungal therapy was reported in 22 patients, accounting for 43 combination treatment episodes (Supplementary Table S4). Empirical antifungal therapy prior to CDC diagnosis was reported for 9/111 patients (8.1%) (Supplementary Table S5).

Considering all treatment switches (n = 201, multiple reasons per switch were possible), modifications were mainly driven by disease progression, including clinical deterioration (n = 52), increasing inflammatory parameters (n = 31), and radiological progression (n = 28) or by disease improvement, including improved inflammatory parameters (n = 50), radiological regression (n = 27), or improved clinical status (n = 27). Oral switch occurred in 37 patients; treatment was discontinued due to death in 18 patients.

The median total duration of targeted treatment was 107 days (IQR 49–182; mean 144.4 days). After treatment discontinuation, the median follow-up was 92 days (IQR 6–361; range 0–3677), during which no CDC relapse was documented. Corticosteroids were administered for suspected immune reconstitution inflammatory syndrome (IRIS) in 5/111 patients (4.5%), characterised by paradoxical radiological progression and persistent fever at a mean of 29 days after CDC diagnosis, typically during neutrophil recovery. Another nine patients received corticosteroids for their underlying haematological disease without clinical suspicion of IRIS.

### Overall survival

**Fig. 4:**
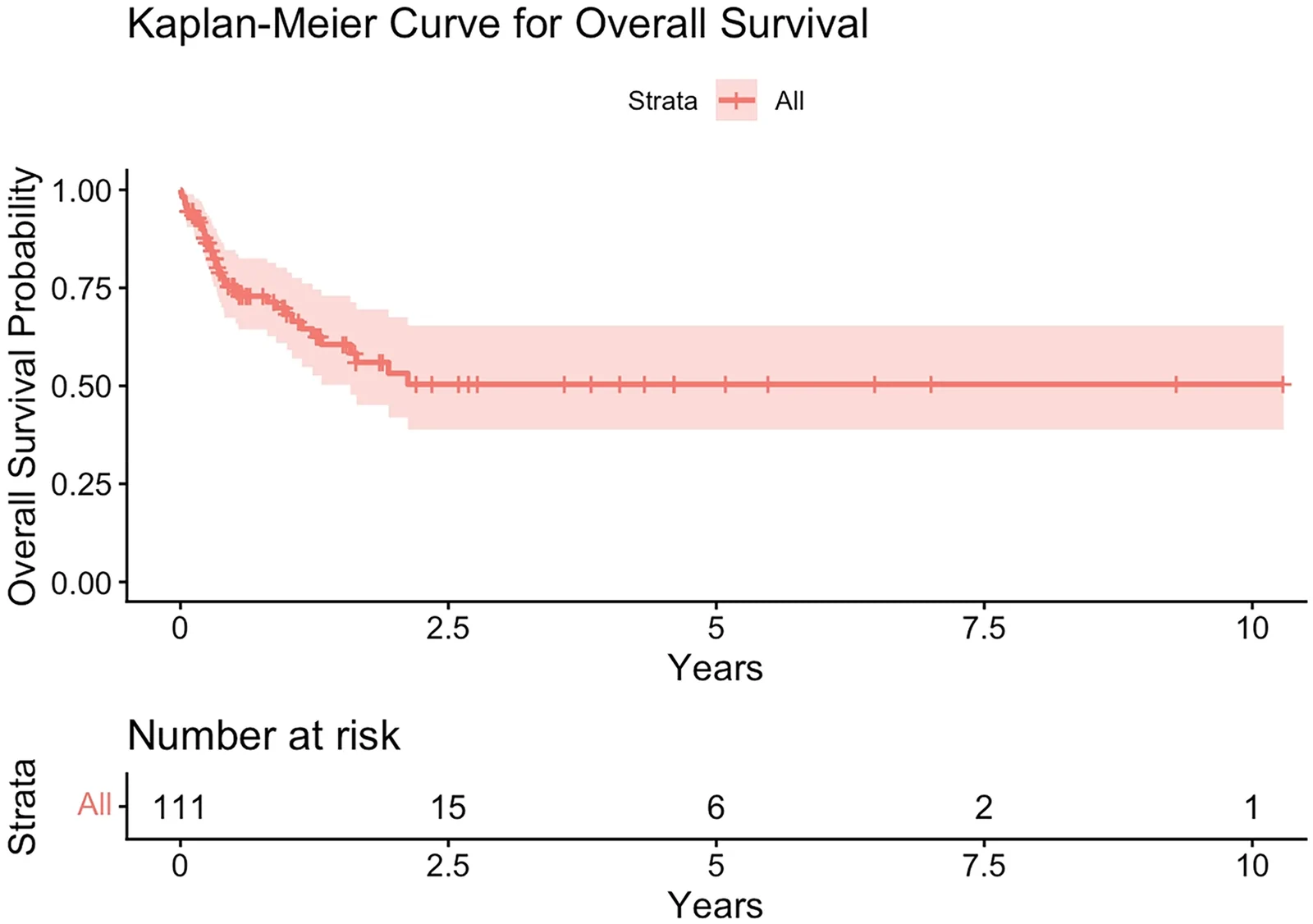
**Kaplan–Meier curve for overall survival**.

At last follow-up, 74/111 patients (66.7%) were alive. The median follow-up was 180 days (IQR 83–562). Most deaths occurred within the first 2.5 years (see Kaplan–Meier analysis; Fig. 4). Death was attributed to CDC in 5/111 patients (4.5%), corresponding to 5/37 (13.5%) of all fatalities. Overall survival was higher in patients with breakthrough CDC compared with non-breakthrough (24/27 [88.9%] vs. 48/82 [58.5%]; p = 0.004). Mortality did not differ significantly between patients with positive blood cultures for *Candida* spp. within the previous 14 days of CDC diagnosis compared to those with negative blood cultures (12/33 [36.4%] vs. 25/78 [32.1%]; p = 0.66), nor between *C. tropicalis* and non-*tropicalis* CDC (9/27 [33.3%] vs. 28/84 [33.3%]; p = 1.00), or between azole-resistant and non-resistant *Candida* strains (3/11 [27.3%] vs. 34/100 [34.0%]; p = 0.75).

**Fig. 5:**
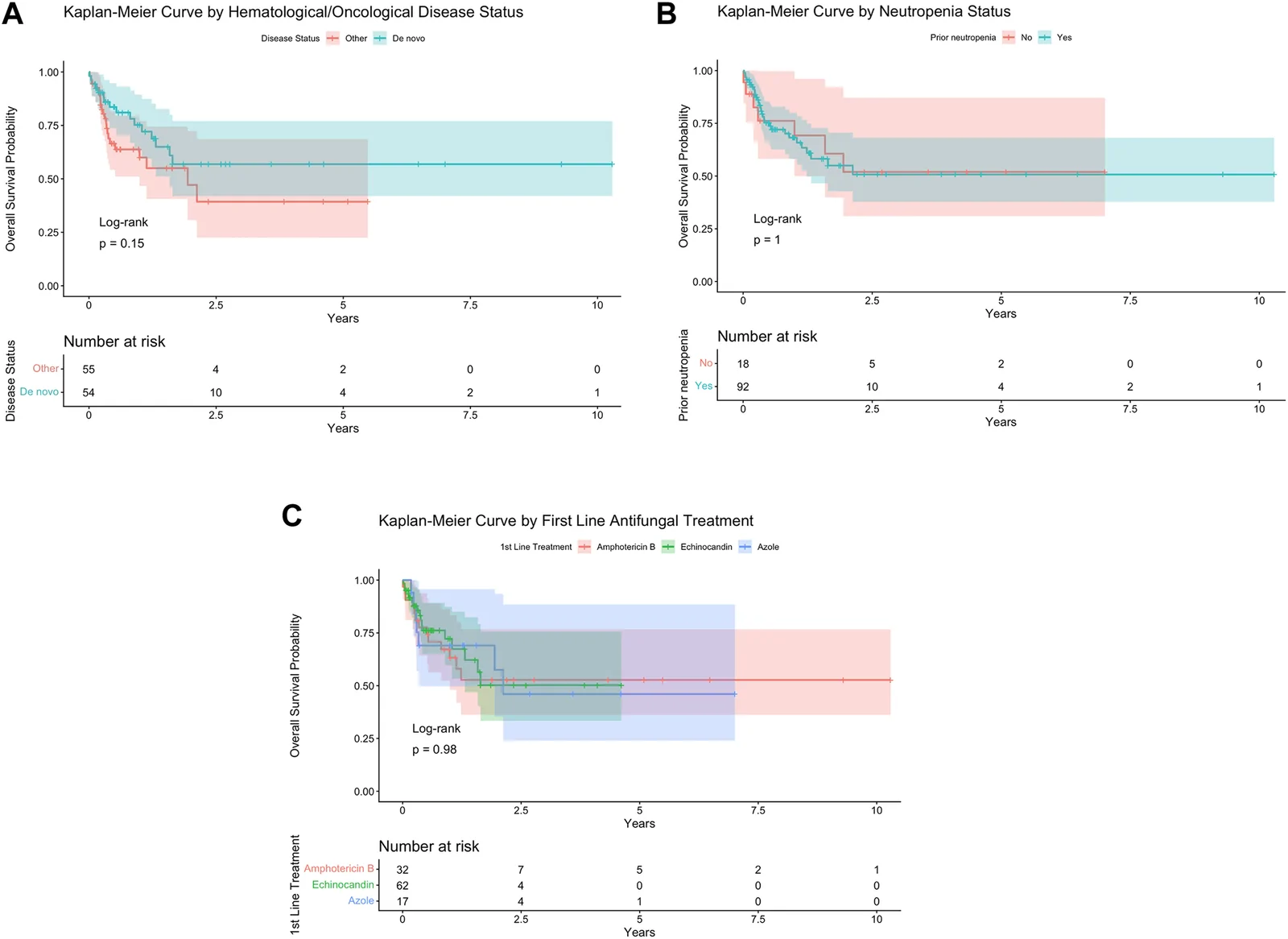
**Kaplan–Meier survival curves according to haematological oncological disease status (A), neutropenia <500 cells/μl for ≥10 days within 30 days prior CDC diagnosis (B) and first-line antifungal treatment group (C)** Survival probabilities were broadly similar between groups, although de novo haematological/oncological disease status showed a trend toward improved survival compared with those with other disease status. Numbers at risk at selected time points are shown below the plot. Amphotericin includes all formulations of amphotericin B (amphotericin B deoxycholate, liposomal amphotericin B, and amphotericin B lipid complex). Estimates beyond the point at which fewer than five patients remain at risk in a given stratum are based on limited follow-up and should be interpreted with corresponding caution.

Survival did not significantly differ between patient groups defined by haematological disease status (de novo vs. other), prolonged neutropenia prior CDC diagnosis or first-line antifungal treatment group (amphotericin B vs. echinocandin vs. azole) (Fig. 5). In the Cox proportional hazards model with amphotericin B as reference, no covariate reached statistical significance. Azole as first-line treatment was associated with a non-significant reduction of overall survival (HR 0.88, 95% CI 0.36–2.18, p = 0.78). Similar results were obtained when including echinocandin as first-line treatment (HR:1.00, 95% CI 0.47–2.13, p = 0.99) (Supplementary Table S6a/b).

### Treatment response

**Fig. 3:**
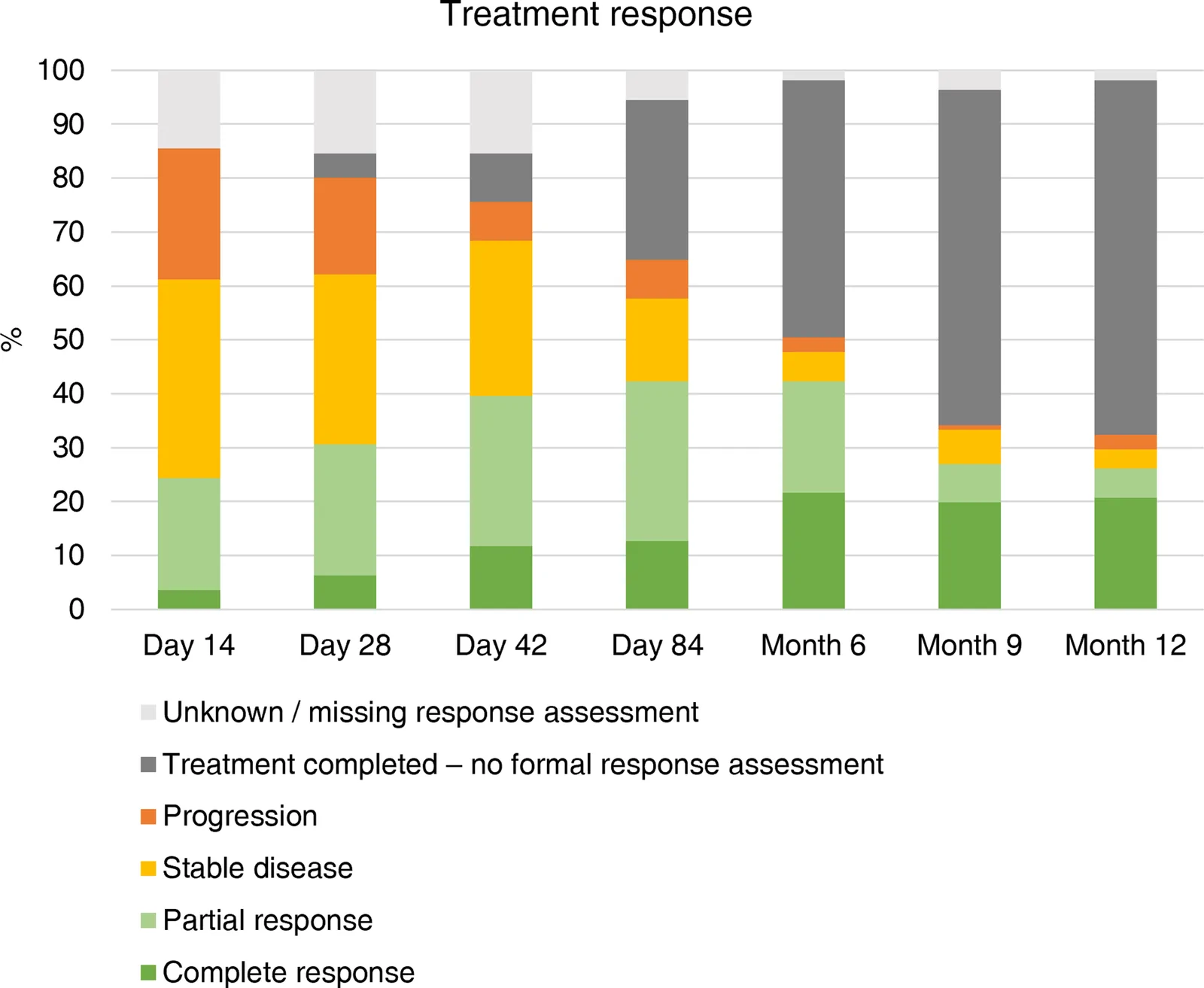
**Treatment response of chronic disseminated candidiasis over time.** Stacked bar chart showing the distribution of treatment response categories. Day 0 is defined as the date of initiation of targeted antifungal therapy for chronic disseminated candidiasis. Treatment response was defined according to a composite evaluation of clinical, radiological and mycological findings. At Day 84, treatment success was achieved in 42.3% of patients (complete response 12.6%, partial response 29.7%), with stable disease in 15.3% and progression in 7.2%. At Month 6, treatment success was 42.3% (complete response 21.6%, partial response 20.7%), with stable disease in 5.4% and progression in 2.7%. Patients who had discontinued antifungal therapy before the respective follow-up time point and for whom no treatment-response assessment was available are shown separately as “Treatment completed - no formal response assessment”. “Unknown/missing response assessment” indicates patients in whom treatment response could not be classified despite ongoing antifungal treatment. Following discontinuation of antifungal therapy, formal treatment-response assessment was recorded only if sufficient information was available; otherwise, follow-up focused on documenting CDC relapse. The numerical data underlying this figure, are provided in Supplementary Table S7a.

Treatment response over 12 months is presented in Fig. 3. The proportions of complete and partial responses increased over time particularly from day 42 onward. At later timepoints (month 6 to month 12), complete response predominated, while progression was infrequent. A sensitivity analysis restricted to patients with proven and probable CDC (n = 85) yielded a similar distribution of treatment responses across all follow-up time points compared with the overall cohort (Supplementary Figure S2 and Supplementary Table S7b). Treatment response (success) at day 84 did not differ significantly across first line antifungal drug classes (amphotericin B 66.7%, echinocandin 68.1%, azole 73.3%, p = 0.90), between breakthrough and non-breakthrough CDC (78.3% vs. 64.5%, p = 0.34), or between proven, probable, and possible cases (59.1% vs. 76.2% vs. 63.6%, p = 0.32). In an exploratory analysis, treatment response did not differ significantly between patients receiving combination antifungal therapy and those without at day 84 (57.1% vs. 72.3%; p = 0.23) or month 6 (78.6% vs. 85.7%; p = 0.68).

In the unweighted mixed-effects logistic regression model including 75 evaluable patients, no statistically significant association between first-line antifungal treatment classes (azoles, echinocandins and amphotericin B) and treatment response between day 84 and 12 months were detected (Supplementary Table S8a). These results remained qualitatively unchanged after adjustment using longitudinal IPTW to account for time-varying confounding for neutropenia and corticosteroids.

In the multivariable IPTW model, baseline BDG positivity (OR 0.21, 95% CI 0.02–2.16), ICU stay (OR 0.16, 95% CI 0.01–2.23), and unknown *Candida* species (OR 0.74, 95% CI 0.16–3.49) all trended towards lower odds of response, though estimates were imprecise owing to the small analytic sample size (Supplementary Table S9a). Univariate IPTW-weighted analyses are presented in Supplementary Table S11a.

First-to-second-line treatment switch analysis showed no significant difference in treatment response at day 84 across the three predefined switch strategies in the propensity score–weighted analysis in the subset of patients entering analysis (Table 3). After initial echinocandin therapy, a switch to fluconazole versus another azole was not significantly associated with treatment response at day 84 (OR 1.04, 95% CI 0.11–9.92; p = 0.97). When considering echinocandins or amphotericin B as first line, switching to fluconazole versus another azole remained not significantly associated with treatment response in this dataset (OR 0.65, 95% CI 0.10–4.37; p = 0.64).

**Table 3:** Propensity score-weighted analysis of first to second-line sequential treatment strategies and treatment response at day 84.

Treatment switch strategy	N	Included in analysis	OR	95% CI	p-value
Strategy A: Initial treatment switch from caspofungin or liposomal amphotericin B					
Caspofungin → fluconazole	8	–	Reference	–	–
Caspofungin → other azoles	7	5	0.55	0.03–9.33	0.655
Liposomal amphotericin B → fluconazole	4	3	NA	–	–
Liposomal amphotericin B → other azoles	11	5	0.57	0.04–9.21	0.668
Strategy B: Initial treatment switch from echinocandin or amphotericin B (drug-classes)					
Echinocandin → fluconazole	11	10	Reference	–	–
Echinocandin → other azoles	12	9	1.04	0.11–9.92	0.972
Amphotericin B → fluconazole	6	4	NA	–	–
Amphotericin B → other azoles	16	8	0.49	0.04–5.41	0.545
Strategy C: Collapsed model from B: Initial treatment switch from echinocandin and amphotericin B					
Echinocandin or amphotericin B → fluconazole	17	14	Reference	–	–
Echinocandin or amphotericin B → other azoles^a^	28	17	0.65	0.10–4.37	0.642

Results from exploratory propensity score-weighted logistic regression models evaluating the association between sequential antifungal treatment strategies (switch from first-line to second-line therapy) and treatment response at day 84. N: total number of patients in each treatment switch group. “Included in analysis”: number of patients contributing to the weighted model. “NA”: non-estimable odds ratios due to complete separation (small subgroups). Given the small subgroup sizes and instances of complete separation, these exploratory analyses should be interpreted with caution.

“Other azoles” refers to azoles excluding fluconazole. Amphotericin B includes all formulations of amphotericin B (deoxycholate, liposomal, and lipid complex). Outcome: binary treatment response at day 84 (complete or partial response vs. stable disease or progression at day 84) imputed from most recent earlier assessment if missing.

Reference for strategy A: Caspofungin → Fluconazole (n = 7). Estimates affected by complete separation (n = 3 in liposomal amphotericin B → fluconazole group).

Reference for strategy B: Echinocandin → Fluconazole (n = 10). Estimates affected by complete separation (n = 4 in amphotericin→ fluconazole group).

Reference for strategy C: Echinocandin/amphotericin B → Fluconazole (n = 14).

OR: odds ratio; CI: confidence interval.

a Including n = 4 breakthrough chronic disseminated candidiasis cases (4/28, 14.3%).

Sensitivity analyses restricted to patients with proven/probable CDC (n = 85), to those with known *Candida* species (n = 72) and to patients without combination therapy (n = 89) yielded findings consistent with the primary analyses (see Supplementary Tables S6c–h; S7b; S8b–d; S9b–c; S10a and b; S11b and c). No statistically significant association between first-line antifungal class and treatment response was observed in either subgroup. ICU stay remained associated with lower treatment response in the *Candida* known-species subgroup but not in the proven/probable subgroup. In the *Candida* species known subgroup, other than “de novo” haematological disease status was associated with lower overall survival (HR 3.63, 95% CI 1.47–8.94; p = 0.005), whereas this association was not observed in the primary or proven/probable analyses.

## Discussion

In this multinational cohort of 111 patients with CDC, no statistically significant associations between first-line antifungal class and treatment response or mortality were detected. Likewise, no statistically significant association was observed between fluconazole and non-fluconazole azoles used for long-term switch and treatment response. These findings remained consistent in sensitivity analyses restricted to patients with proven/probable CDC, to those with known *Candida* species, and to those without combination therapy, where estimable.

The predominance of AML and ALL in this cohort is consistent with series from comparable periods^2^, ^3^, ^4^^,^^7^ whereas earlier studies reported lower rates of ALL or lymphoma,^5^^,^^21^ possibly reflecting the increased use of azole prophylaxis in AML and intensified immunosuppressive treatment regimens in lymphoma. In this cohort, the higher rate of probable and proven CDC compared to other series^3^^,^^7^ likely reflects increased availability of BDG testing and reporting bias. The observed rate of prior candidaemia however was similar to previous reports.^2^^,^^21^

CNS involvement has not been systematically reported in previous CDC cohorts. Although the identification of CNS involvement in nine patients may partly reflect a selection bias and increased diagnostic awareness within participating centres, the consistent imaging findings and cerebrospinal fluid BDG positivity observed in several cases suggest that CNS dissemination may be under-recognised. These observations support consideration of CNS imaging in selected patients with neurological symptoms or refractory CDC and are consistent with previous reports of CNS candidiasis described in haematological populations.^22^

*C. albicans* (32.4%) and *C. tropicalis* (24.3%) accounted for the majority of identified isolates, with a marked geographical clustering of *C. tropicalis* in Taiwan and Colombia, consistent with previously reported geographic variation.^3^^,^^4^^,^^7^ *C. tropicalis* was associated with cutaneous dissemination in most cases, as previously described,^23^ and should be considered in the differential diagnosis in neutropenic patients. Fluconazole resistance occurred more frequently among *C. tropicalis*, in line with surveillance data^16^ and frequently occurred in the context of breakthrough CDC. These findings extend the limited available evidence by comparing *Candida* species distribution and associated fluconazole resistance patterns. The proportion of breakthrough CDC (24.8%) was similar to that reported in other contemporary studies.^2^^,^^4^ Patients with breakthrough CDC had higher crude overall survival than those without breakthrough infection (88.9% vs 58.5%). Although our data cannot explain the biological mechanisms underlying this observation, the low CDC-attributable mortality (4.5%) suggests that host-related factors, including the underlying haematological disease status at CDC diagnosis, may be more important determinants of prognosis than breakthrough infection itself. However, this interpretation is limited by data heterogeneity, the observational design, residual confounding, and the absence of survival comparisons between breakthrough and non-breakthrough cases in previous studies.

Early progression at first follow-up imaging was common (37.8% at 14 days) and likely reflects paradoxical worsening during neutrophil recovery rather than antifungal treatment failure. As imaging lags behind clinical improvement and conventional modalities do not capture metabolic activity, residual lesions could skew towards overtreatment as previously highlighted.^24^ PET/CT was performed in 29 (26.1%) of patients, predominantly in prolonged or clinically complex cases. Regression of findings directly informed treatment discontinuation in 12 patients, illustrating its emerging role in individualised CDC management despite only limited recommendations in current guidelines.^9^^,^^13^ Although observational and subject to confounding by indication as well as differences in PET/CT availability across participating centres, these findings complement the prospective multicentric CANHPARI study, supporting PET/CT-guided individualised treatment decisions after 3 months of antifungal therapy.^9^

Supporting this approach, the median antifungal treatment duration in this cohort was 107 days, whereas previous cohorts reported longer treatment durations^18^^,^^21^ and no CDC relapses were observed following treatment discontinuation. Together, these findings suggest that antifungal treatment duration should be individualised rather than extended until complete radiological resolution in selected patients. These findings are hypothesis-generating and require prospective confirmation before informing treatment duration in routine practice.

The observed BDG sensitivity (88.6%) was higher than previously reported (∼50%),^2^^,^^10^ which may reflect reporting and selection bias, and the retrospective inclusion of investigator-submitted, predomantly well-characterised cases. Persistently positive BDG (≥28 days) was explored but could not be robustly modelled because serial measurements were available for only a small subset of patients. Future prospective studies with standardised longitudinal BDG sampling are needed to validate the prognostic value suggested by small retrospective studies.^10^ The rate of paradoxical worsening requiring adjunctive corticosteroids was low (4.5%), whereas reported rates in other studies are heterogeneous.^2^^,^^4^^,^^25^^,^^26^ This reflects the ongoing uncertainty regarding the role of corticosteroids in CDC-IRIS^27^ and underscores individual immunological factors as key determinants of CDC disease evolution.^28^^,^^29^ More studies are needed to evaluate individualised immunomodulatory strategies, including corticosteroids, interferon-γ, and immune checkpoint inhibitors, in carefully selected patients.

All-cause mortality was 33.3%, with CDC-attributable mortality of only 4.5%, which is consistent with prior reports suggesting that outcomes are largely driven by the underlying haematological malignancy rather than CDC itself.^2^^,^^4^^,^^7^ In this cohort, no statistically significant associations between first-line antifungal class, candidaemia, or *Candida* species and overall survival were detected suggesting that host-related factors may have a greater influence on prognosis than pathogen-related characteristics.^2^ Likewise, no statistically significant association between antifungal resistance, including fluconazole resistance among *C. tropicalis* isolates, and clinical outcomes was detected. However, the exploratory nature of these analyses precludes firm conclusions.

Echinocandins were the most commonly used first-line treatments (65/111, 58.6%), followed by polyenes (33/111, 29.7%), reflecting a shift from earlier cohorts in which azoles or polyene predominated,^2^^,^^4^^,^^5^^,^^7^ and aligning with current guidelines and evidence.^12^^,^^13^^,^^30^ In this dataset, no first-line antifungal class (azoles, echinocandins, or polyenes) was significantly associated with improved treatment responses between day 84 and month 12 or survival, even after accounting for time-dependent confounders neutropenia and corticosteroid exposure which were not considered in prior studies. This is in contrast to previous studies that suggested superiority of either echinocandins or liposomal amphotericin B as first-line therapy.^4^^,^^5^ Treatment response tended to be lower in patients with positive BDG at diagnosis, ICU stay, and unknown *Candida* species; though none of these associations reached statistical significance. We note, that these analyses were exploratory, with a small effective sample size in adjusted models and substantial missing data, limiting interpretability. Previously reported predictors of treatment failure, including prior prolonged neutropenia or proven CDC^4^ were not confirmed in this cohort.

Treatment modifications were frequent, with a transition from first- to second-line treatment, most commonly from echinocandins to azoles. Switches beyond second-line therapy, including multiple sequential regimens and combination antifungal therapy, were also common, likely reflecting the complexity of CDC response assessment and diagnostic uncertainty arising from overlap with IRIS, underlying malignancy progression, or concurrent infections. In the propensity score–weighted analysis including a subset of 26 patients, switching from echinocandins or polyenes to fluconazole was not associated with a statistically significant difference in treatment response compared with switching to other azoles. Although exploratory, these findings provide the first comparative data on long-term azole step-down strategies and other treatment switches in CDC, an area for which current guidelines acknowledge a lack of evidence.

This study has several strengths, including the largest cohort to date, a multinational design, and the first incorporation of time-dependent variables, as well as analysis of treatment switch strategies in CDC. However, the study is limited by its retrospective design, heterogeneity in diagnostic practices and diagnostic availability, temporal changes in clinical practice, and variability in treatment access across centres. Furthermore, treatment response was assessed as a single, non-adjudicated investigator judgement, limiting the objectivity and standardisation of outcome assessment and introducing the potential for inter-centre variability. Similarly, reasons for treatment modification were recorded using predefined categories applied by treating investigators at each site without a standardised cross-centre operational definition; these categories may therefore not be fully comparable across participating centres. Given the wide variation in per-country sample size, country- or centre-level clustering could not be modelled as a covariate or random effect without introducing model instability; cross-country differences in antifungal prophylaxis strategy, haematological treatment regimens, and follow-up protocols therefore remain unmeasured sources of potential confounding. Because cases were identified through investigator-submitted reports following network outreach rather than systematic, population-based case ascertainment, and because no population denominator was available, this cohort cannot be used to estimate the incidence of CDC, temporal trends, or geographic variation in disease occurrence. No control group, central blinded radiological review, or standardised cross-centre PET/CT assessment protocol was implemented. PET/CT was not performed systematically and was mainly performed for patients with prolonged disease courses, limiting conclusions regarding its comparative clinical utility. Antifungal dosing, therapeutic drug monitoring, and renal and hepatic function were not systematically captured and therefore could not be accounted for in the comparative treatment analyses. The impact of empirical and combination antifungal therapy could not be adequately assessed due to considerable treatment heterogeneity and small sample size. In addition, residual confounding, selection and reporting bias, the small sample size in the IPTW analysis, and the omission of immunosuppression as a time-dependent variable due to model instability further limit the interpretability and generalisability of the findings. Furthermore, long-term follow-up after treatment discontinuation was heterogeneous, and late CDC relapse, long-term antifungal toxicity, oncological outcomes, and health-economic endpoints were not systematically captured, limiting conclusions regarding the long-term benefit–risk profile of individualised treatment duration.

This study expands the contemporary evidence base for the management of CDC by providing the first multinational comparative evaluation of antifungal treatment strategies, long-term treatment modification, PET/CT-guided management in routine clinical practice.

Collectively, these exploratory analyses did not identify evidence supporting the superiority of any antifungal strategy after accounting for time-varying confounding, providing a contemporary framework for individualised CDC management while highlighting the need for prospective comparative studies. These findings support an individualised approach to treatment and highlight the need for more objective tools to assess treatment response and optimise treatment duration. Given the rarity of CDC, future prospective international studies should incorporate standardised treatment algorithms, PET/CT-guided response assessment with predefined timing and response criteria, candidate biomarkers of host immune reconstitution already piloted in smaller cohorts,^28^^,^^29^ including cytokine profiles and *Candida* antigen-specific T-cell responses, and serial BDG measurement, embedded within a registry-based platform to overcome the sample-size constraints inherent to this rare disease. Such collaborative efforts will be essential to improve individualised management and to evaluate novel antifungal agents and adjunctive immunomodulatory therapies, particularly for patients where the use of currently established treatment options are limited.

## Contributors

IR and DS conceptualised the study, curated the data, conducted the formal analysis and investigation, developed the methodology, administered the project, validated the findings, prepared the visualisations, drafted the original manuscript, and contributed to reviewing and editing the final version. IR, DS, and DSB conducted the formal analysis. IR, DS, AV, MVM, and RS developed the methodology. IR and ES validated the findings. IR, ES and DS accessed and verified the underlying data. IR, DS, LH, and DSB were responsible for visualisation. IR, DS, and DSB wrote the original draft of the manuscript. IR, DS, SS, PK, IRC, IRa, PYC, YC, ADh, JL, SA, MB, CG, SAI, ADP, SK, DAZ, AB, AC, SE, JG, JI, SJ, RK, DL, GAM, JAN, MN, GS, SS, JS, KA, DAE, DF, OJ, PKo, RK, JM, FM, CO, NS, YV, NW, LH, JH, JP, ES, JS, DSB, RS, MVM, AV, LP, OAC, and DS reviewed and edited the manuscript. All authors read and approved the final version of the manuscript. IR, DS and OAC were responsible for the decision to submit the manuscript for publication.

## Data sharing statement

The data that support the findings of this study are available from the corresponding author upon reasonable request, subject to institutional and ethical approvals.

## Declaration of interests

IR received a personal grant from the Gusyk Förderung from the University of Cologne, Germany to support the submitted work, speakers fee from Menarini Group. IRa reports receiving honoraria for lectures from Knight Therapeutics; support for attending meetings from Knight Therapeutics. YC reports receiving funding support from the Ministry of Health and Welfare, Taiwan (MOHW113-TDU-B-211-114006) for the submitted work. SA reports receiving grants or contracts, consulting fees, honoraria and board participation from Napp/Mundipharma, Gilead Sciences, Shionogi. SAI reports receiving support for attending meeting from Gilead. DAZ reports receiving honoraria as a speaker from Knight Therapeutics and Gilead. SE reports receiving honoraria for lectures, presentations, speakers bureaus, manuscript writing or educational events from Gilead Sciences. SS reports receiving consulting fees, travel support and honoraria for lectures, presentations, speakers bureaus, manuscript writing or educational events from Pfizer. JSM received honoraria for lectures, presentations, speakers bureaus, manuscript writing or educational events from Gilead, Pfizer. DE received funding support for the submitted work, grants or contracts, payment for presentation and support for attending meeting from Mundipharma. PK reports institutional grants or contracts from German Federal Ministry of Research and Education (BMBF), B-FAST (Bundesweites Forschungsnetz Angewandte Surveillance und Testung) and NAPKON (Nationales Pandemie Kohorten Netz, German National Pandemic Cohort Network) of the Network University Medicine (NUM), the State of North Rhine-Westphalia and the Dr. Heinz Lux-Stiftung; Consulting fees from Ambu GmbH, Gilead Sciences, Mundipharma Research Limited, Noxxon N.V. and Pfizer Pharma; Honoraria for lectures from Akademie für Infektionsmedizin e.V., Ambu GmbH, Astellas Pharma, BioRad Laboratories Inc., European Confederation of Medical Mycology, Gilead Sciences, GPR Academy Ruesselsheim, HELIOS Kliniken GmbH, Jazz Pharmaceuticals Germany GmbH, medupdate GmbH, MedMedia GmbH, MSD Sharp & Dohme GmbH, Pfizer Pharma GmbH, Sanofi-Aventis Deutschland GmbH, Scilink Comunicación Científica SC and University Hospital, LMU Munich, Verein zur Förderung der Rheumatologie e.V.; Travel support from Sanofi-Aventis Deutschland GmbH, European Confederation of Medical Mycology and Pfizer Pharma GmbH; Participation on an Advisory Board from Ambu GmbH, Duke University, Gilead Sciences, Pfizer Pharma, Mundipharma Research Limited, Novartis Pharma GmbH, Noxxon N.V. and VITIS GmbH; A filed patent at the German Patent and Trade Mark Office (DE 10 2021 113 007.7); Other non-financial interests from Elsevier, Wiley and Taylor & Francis online outside the submitted work. JM reports receiving consulting fees, honoraria and board participation from F2G, Mundipharma, Basilea, Gilead Sciences, Takeda, Elion. NS reports receiving consulting fees from Napp/Mundipharma; Speaker fees from Hikma, Gilead, Pfizer, Napp/Mundipharma; Support for conference from Hikma Pharmaceuticals. YV reports receiving institutional scientific grants from Gilead, “Kom op Tegen Kanker” from a Belgian non-profit organisation supporting cancer research, “KOOR FAS” from UZ Leuven; Speaker fees from iInfluence; Support for attending congress and travel support from Pfizer, Gilead, Neovii, Alexion, Menarini; Receipt of equipment, materials, drugs, medical writing, gifts or other services from PathoNostics, Pearl Diagnostics. JP reports receiving consulting fees and honoraria from MSD and Pfizer; leadership or fiduciary role from DGHO and AGIHO. JS reports receiving institutional grants or contracts from the German Federal Ministry of Education and Research, Medical Faculty of the University of Cologne, Basilea Pharmaceutica, Noscendo, Scynexis; Consulting fees from AiCuris, Alvea Vax, Gilead, Mundipharma; Payment or honoraria from AbbVie, Akademie für Infektionsmedizin, Forum für Medizinfortbildung, Eli-Lilly, Hikma, Mundipharma, Pfizer; Payment for expert testimony from Shionogi; Travel/Meeting support from Page Medical, Shionogi; Board participation from Kite-Gilead, Micron Research; Leadership or fiduciary role from Infectious Disease Working Party of the German Society for Hematology and Medical Oncology. RS reports receiving grants or contracts from Deutsches Zentrum für Infektionsforschung, Ministerium für Kultur und Wissenschaft des Landes Nordrhein-Westfalen; Honoraria from Pfizer, Akademie für Infektionsmedizin e.V., Hikma, Mundipharma, FomF, Ärztliche Akademie für medizinische Fort-und Weiterbildung in Nordrhein, Infektio Saar Netz; Travel/Meeting support from ECMM, Pfizer, Page Medical, ESCMID, ISHAM; leadership or fiduciary role from Young ECMM, DMykoG Foundation, CPAnet. MVM reports receiving honoraria from Pfizer, MSD, ViiV, Menarini, Gilead. AV reports receiving payment or honoraria from Pfizer, Gilead, Advanz Pharma, Shionogi, Menarini, Tillotts Pharma; Travel support from Pfizer, Gilead; Board Participation from Pfizer, Merck, Advanz Pharma, Shionogi. OAC reports institutional grants or contracts from AiCuris, AstraZeneca, Basilea, BMFTR, Cidara, DFG, DZIF, EU-DG RTD, F2G, G-BA, Gilead, iMi, iHi, IQVIA, MedPace, Melinta, MSD, Moderna, Mundipharma, Noscendo, Octapharma, Pfizer, Scynexis, Vedanta; Consulting fees from Abbvie, AiCuris, Aurobac, Basilea, Cidara, Elion, Gilead, GSK, IQVIA, Janssen, MedPace, Menarini, Melinta, Mundipharma, Octapharma, Pardes, Pfizer, PSI, Scynexis, Seqirus, Shionogi; Speaker and lecture honoraria from Abbott, Abbvie, Hikma, AstraZeneca, Asahi Kasei, Gilead, GSK, Knight, InfectoPharm, Ipsen, Medscape/WebMD, MSD, Moderna, Mundipharma, Noscendo, Paul-Martini-Stiftung, Pfizer, Sandoz, Seqirus, Shionogi, Universimed, Vitis; Participation on a DRC, DSMB, DMC, or advisory board for AstraZeneca, Cidara, IQVIA, Janssen, MedPace, Melinta, PSI, Pulmocide, Vedanta. All other authors do not report any competing interests related to this manuscript.
