# Supplementary material for: Treatment and outcome of chronic disseminated candidiasis in the era of antifungal prophylaxis: a retrospective multicentre cohort of 111 patients
Source: eClinicalMedicine. 2026 Sep 16;100:104194. doi: 10.1016/j.eclinm.2026.104194 (PMC13595010; doi:10.1016/j.eclinm.2026.104194)
Supplement: Supplementary file 1 [file mmc1.pdf]

Supplementary appendix

Table of Contents

Supplementary Methods 4
Scientific societies implemented in outreach and call for cases 4
Collected data within the electronic case report form 4
Antifungal susceptibility testing. 4
Patient identification and data collection, case ascertainment and case exclusion 4
Statistical analysis 4
Supplementary Results 6
Supplementary Table S1. Standardized treatment response definitions incorporated into the electronic case report form 6
Supplementary Table S2. Country of origin of patients with chronic disseminated candidiasis reported as case contribution 7
Supplementary Figure S1. Year of diagnosis of 111 chronic disseminated candidiasis cases 8
Supplementary Table S3. Underlying haematological/oncological disease compared between breakthrough and non-breakthrough chronic disseminated candidiasis 9
Antifungal susceptibility testing methods. 9
Supplementary Table S4. Combination (overlapping) antifungal therapy for more than 3 days during treatment courses in 22 patients with CDC accounting for 43 overlapping episodes 10
Supplementary Table S5. Empirical antifungal therapy in nine patients with chronic disseminated candidiasis 11
Supplementary Table S6a. Cox proportional hazards model for overall survival (replication of Boussen et al.) [1] 12
Supplementary Table S6b. Cox proportional hazards model for overall survival including echinocandin as first-line treatment 12
Supplementary Table S6c (sensitivity analysis). Cox proportional hazards model for overall survival (replication of Boussen et al.) — proven/probable cohort (n=85) 13
Supplementary Table S6d (sensitivity analysis). Cox proportional hazards model for overall survival including echinocandin as first-line treatment — proven/probable cohort (n=85) 13
Supplementary Table S6e (sensitivity analysis). Cox proportional hazards model for overall survival (replication of Boussen et al.) — known *Candida* species cohort (n=72) 14
Supplementary Table S6f (sensitivity analysis). Cox proportional hazards model for overall survival including echinocandin as first-line treatment — known-*Candida* species cohort (n=72) 14
Supplementary Table S6h (sensitivity analysis). Cox proportional hazards model for overall survival including echinocandin as first-line treatment — cohort without combination therapy (n=89) 15
Supplementary Table S7a. Treatment response over time (n = 111). 16
Supplementary Figure S2. Treatment response over time in patients with proven or probable chronic disseminated candidiasis (n=85; sensitivity analysis). 17
Supplementary Table S7b. Treatment response over time in the sensitivity cohort restricted to proven and probable chronic disseminated candidiasis (n = 85). 17
Supplementary Table S8a. Unweighted mixed-effects logistic regression model for treatment response day 84 through month 12. 18
Supplementary Table S8b (sensitivity analysis). Unweighted mixed-effects logistic regression model for treatment response day 84 through month 12 — proven/probable cohort 18

Supplementary Table S8c (sensitivity analysis). Unweighted mixed-effects logistic regression model for treatment response day 84 through month 12 — known Candida species cohort ... 19

Supplementary Table S8d (sensitivity analysis). Unweighted mixed-effects logistic regression model for treatment response day 84 through month 12 — cohort without combination therapy ... 19

Supplementary Table S9a. IPTW-weighted marginal structural model adjusted for baseline covariates (BDG, ICU stay, unknown *Candida* species) for treatment response day 84 to month 12. ... 20

Supplementary Table S9b (sensitivity analysis). IPTW-weighted marginal structural model adjusted for baseline covariates (BDG, ICU stay, unknown Candida species) for treatment response day 84 to month 12 — proven/probable cohort ... 20

Supplementary Table S9c (sensitivity analysis). IPTW-weighted marginal structural model for treatment response day 84 to month 12 — known *Candida* species cohort ... 21

Supplementary Table S9d (sensitivity analysis). IPTW-weighted marginal structural model for treatment response day 84 to month 12 — cohort without combination therapy ... 21

Supplementary Table S10a (sensitivity analysis). Propensity score-weighted analysis of first to second-line sequential treatment strategies and treatment response at day 84 months — proven/probable cohort ... 22

Supplementary Table S10b (sensitivity analysis). Propensity score-weighted analysis of first to second-line sequential treatment strategies and treatment response at day 84/3 months — known *Candida*-species ... 23

Supplementary Table S10c (sensitivity analysis). Propensity score-weighted analysis of first to second-line sequential treatment strategies and treatment response — cohort without combination therapy ... 24

Supplementary Table S11a. Univariate IPTW-weighted models for treatment response (day 84 through month 12) — baseline covariates. ... 26

Supplementary Table S11b (sensitivity analysis). Univariate IPTW-weighted models for treatment response (day 84 through month 12) — baseline covariates, proven/probable cohort ... 27

Supplementary Table S11c (sensitivity analysis). Univariate IPTW-weighted models for treatment response (day 84 through month 12) — baseline covariates, known *Candida* species cohort ... 29

Supplementary Table S12. Comparison of the present cohort with previously published chronic disseminated candidiasis / hepatosplenic candidiasis series, among the largest reported to date. ... 31

References ... 33

Supplementary Methods

Scientific societies implemented in outreach and call for cases

- European Confederation of Medical Mycology (ECMM)
- ESCMID (European Society of Clinical Microbiology and Infectious Diseases) Fungal Infection Study Group (EFISG): endorsement
- ESCMID Study Group for Infections in Compromised Hosts (ESGICH): endorsement
- German-speaking Mycological Society (DMykG)

Collected data within the electronic case report form

Collected data included patients´ demographics; underlying haematological disease and treatment; neutropenia; mucositis; prior exposure to corticosteroids, antibiotics, immunosuppressives, and antifungal prophylaxis. Characteristics of CDC were described by sites of infection; imaging modalities; laboratory parameters including liver values and BDG; mycological evidence; *Candida* species and antifungal susceptibility when available; antifungal treatment regimens and reasons for modification or discontinuation; and use of immunomodulatory therapies in case of paradoxical worsening or suspicion of immune reconstitution inflammatory syndrome (IRIS).

Antifungal susceptibility testing.

Antifungal susceptibility testing was performed locally at participating centres according to routine clinical practice and was not standardized across the registry. Where available, the testing methodology reported in the electronic case report form (eCRF) included Sensititre YeastOne, Vitek 2, EUCAST broth microdilution, CLSI broth microdilution, and E-test. For the present analyses, fluconazole resistance was classified according to the susceptibility interpretation reported by the local laboratory. Only isolates reported as resistant (R) were classified as resistant in the dichotomous resistance analyses; isolates reported as susceptible, increased exposure (I) were not classified as resistant.

Patient identification and data collection, case ascertainment and case exclusion

Collaborating investigators were asked to consider all patients at their site potentially meeting the CDC case definition, however, no independent completeness audit was performed per site. Where classification as proven, probable, or possible CDC was uncertain, additional clinical, radiological, and microbiological detail was requested from the patient's medical chart before a case was proposed for inclusion. In addition, a number of CDC cases (n=20) already existing within the FungiScope® registry before the dedicated call for cases, were identified retrospectively and re-reviewed regarding meeting definition criteria. Every newly submitted case was reviewed centrally by the study team and additionally by a single investigator (IR), prior to case validation; this process also served to identify potential duplicate submissions across the geographically dispersed participating centres. Cases were excluded before entering the database where the minimum case definition could not be confirmed or, in a subset, where imaging showed findings not characteristic of chronic disseminated candidiasis (e.g., a solitary liver lesion). Nine cases entered in the database were excluded due to missing information on antifungal treatment data (n=2), failure to meet definition criteria (n=7), and one case of disseminated *Geotrichum candidum* infection resulting in 111 cases for analysis.

Statistical analysis

To mirror the previously published analysis by Boussen et al.[1], a Cox proportional hazards model was fit including baseline azole, adjunctive corticosteroid administration, age at diagnosis, neutropenia within 30 days, and haematologic disease state (de novo vs. other) at diagnosis as covariates, with amphotericin as reference treatment. In a second Cox proportional hazard model echinocandin was also included.

To evaluate treatment response over time, marginal structural modelling was used to address time-varying confounding. Stabilized inverse probability of treatment weights were estimated using “WeightIt” (Greifer N. (2025). WeightIt: Weighting for Covariate Balance in Observational Studies. R package version 1.5.1, https://CRAN.R-project.org/package=WeightIt.) for dominant antifungal class (azole, echinocandin, polyene, or no treatment, reference: no treatment) using longitudinal multinomial treatment models, with corticosteroid exposure, neutrophil count (< or ≥ 500cells/μL), included in the denominator model. Immunosuppression as a time-dependent covariate could not be retained in the model due to instability, related to sparse events and limited variability over time. Weighted outcome models were then fit using survey-weighted generalized linear models with quasibinomial errors, via “survey” (T. Lumley (2004). Analysis of complex survey samples. Journal of Statistical Software 9(1), 1-19. https://doi.org/10.18637/jss.v009.i08). Response was assessed at four timepoints only (day 84, month 6, month 9, and month 12); timepoints prior to day 84 were excluded as too early to reflect treatment-attributable effects on response.

To explore baseline confounding, candidate baseline covariates were added individually to the weighted model (Supplementary Table S11a). Resulting p-values were adjusted for multiple testing using the Benjamini-Hochberg procedure. Owing to sparse data and quasi-separation for some variables, the final multivariable weighted response model retained only the baseline covariate considered estimable and clinically interpretable.

Separate analyses were performed for antifungal treatment-switch strategies among patients who underwent a first-to-second line treatment change. For these analyses, the primary outcome was treatment response at day 84. If day 84 response was missing, it was imputed from the most recent earlier response assessment in the sequence day 42, day 28, day 14.

Three predefined switch variables were examined: Variable 1) drug-specific switch strategy: caspofungin→fluconazole; liposomal amphotericin B→fluconazole; caspofungin→other azole; liposomal amphotericin B →other azole); Variable 2) class-level switch strategy: a class-level variable (echinocandin→fluconazole; any formulation of amphotericin B →fluconazole; echinocandin→other azole; any formulation of amphotericin B →other azole); and 3) a binary collapsed variable (echinocandin or any formulation of amphotericin B→fluconazole vs. echinocandin or amphotericin B→other azole). In patients receiving combination antifungal therapies, classification was based on the antifungal agent administered for the longest duration.

For each switch analysis, stabilized propensity-score weights for the multinomial switch exposure were estimated using time to treatment switch, age at diagnosis, corticosteroid administration, neutrophil count at time of switch (<500 vs. ≥500 cells/μL), immunosuppression status at time of switch, reason for switch (improvement vs. worsening; based on a composite assessment of clinical, radiological and inflammatory parameters, including switch to oral therapy as a proxy for clinical improvement), intensive care unit stay, and unknown *Candida* species as covariates, using “WeightIt” (Greifer, 2025).

Weighted binary response models were fit using survey-weighted logistic regression. These included time to treatment switch from first to second line, age at diagnosis as additional covariates. Global significance of the switch variable was assessed with likelihood ratio testing.

To visualize treatment pathways, Sankey diagrams were created for sequential antifungal therapies using “networkD3” (Allaire J, Gandrud C, Russell K, Yetman C (2025). networkD3: D3 JavaScript Network Graphs from R. R package version 0.4.1, https://CRAN.R-project.org/package=networkD3).

Supplementary Results

Supplementary Table S1. Standardized treatment response definitions incorporated into the electronic case report form

**Response category**	**Definition**
Complete response	Resolution of all attributable signs and symptoms of disease, radiological abnormalities, and mycological evidence of eradication of disease.
Partial response	Improvement in attributable signs and symptoms of disease, radiological abnormalities, and evidence of clearance of cultures or reduction of fungal burden.
Stable disease	Minor or no improvement in fungal disease but no evidence of progression, based on clinical, radiological, and mycological findings.
Progression	Evidence of progressive fungal disease based on a composite of clinical, radiological, and mycological findings.
Unknown	Insufficient information available to classify treatment response.

Treatment response was assessed relative to Day 0 (start of targeted antifungal treatment for chronic disseminated candidiasis) at day 14, day 28, day 42, day 84, and after 6, 9, and 12 months using the standardized response definitions incorporated into the electronic case report form.

Supplementary Table S2. Country of origin of patients with chronic disseminated candidiasis reported as case contribution

Country	Number of cases	%
Germany	25	22.5
Taiwan	13	11.7
Colombia	11	9.9
France	10	9.0
United Kingdom	10	9.0
Italy	7	6.3
Spain	7	6.3
Russian Federation	5	4.5
Serbia	4	3.6
Argentina	3	2.7
Japan	3	2.7
Turkey	3	2.7
Egypt	2	1.8
Mexico	2	1.8
Portugal	2	1.8
Austria	1	0.9
Belgium	1	0.9
Nigeria	1	0.9
Switzerland	1	0.9

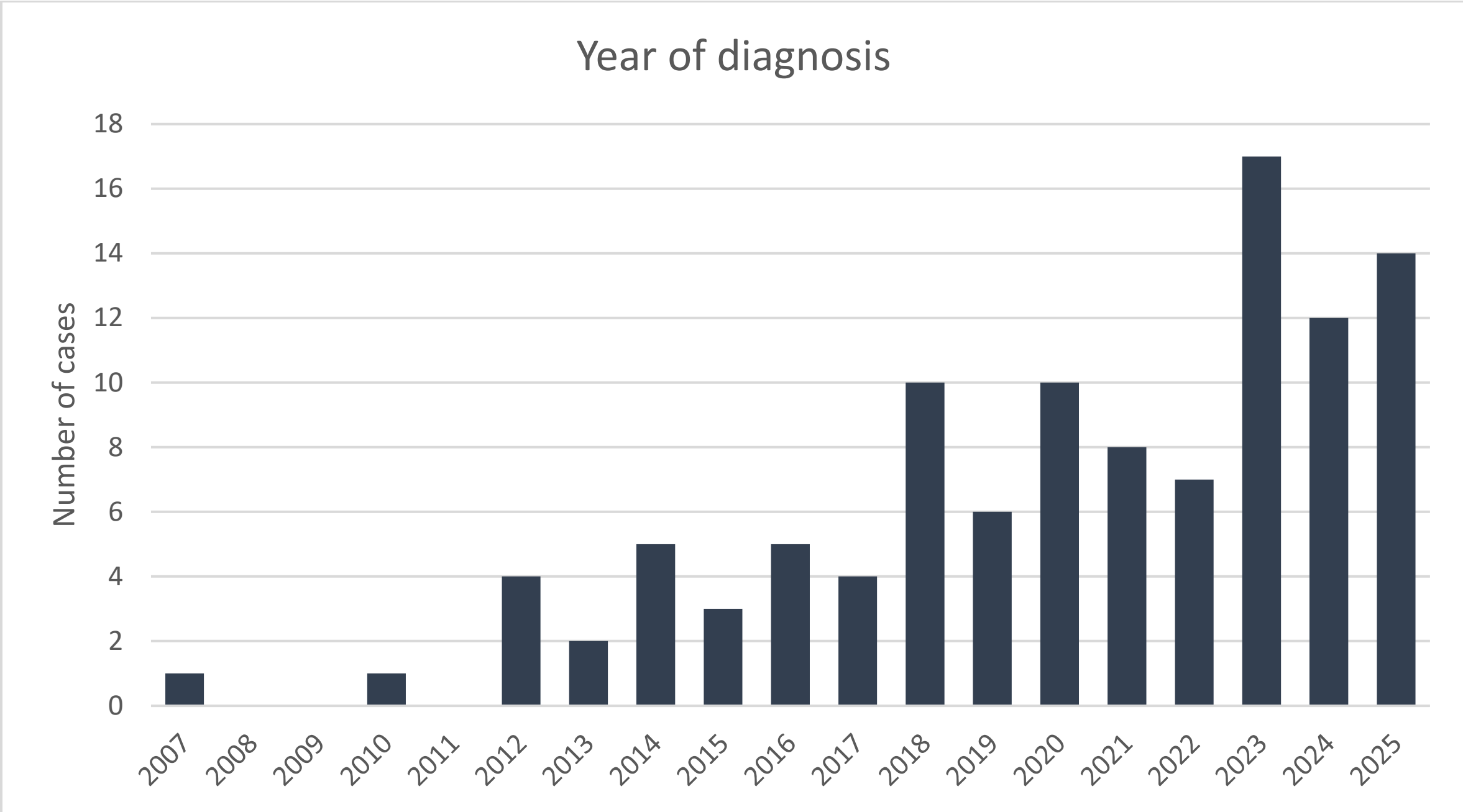

Supplementary Figure S1. Year of diagnosis of 111 chronic disseminated candidiasis cases

Supplementary Table S3. Underlying haematological/oncological disease compared between breakthrough and non-breakthrough chronic disseminated candidiasis

Disease	Non-breakthrough CDC N=82 n (%)	Breakthrough CDC N=27 n (%)	OR	p-value
Acute myeloid leukaemia	43 (52.4)	10 (37.0)	1.87	0.188
Acute lymphocytic leukaemia	22 (26.8)	5 (18.5)	1.61	0.451
Lymphoma	11 (13.4)	6 (22.2)	0.54	0.358
Myelodysplastic syndrome	2 (2.4)	3 (11.1)	0.20	0.096
Multiple myeloma	1 (1.2)	1 (3.7)	0.32	0.436
Chronic lymphocytic leukaemia	1 (1.2)	0 (0.0)	—	1.000
Chronic myelogenous leukaemia	0 (0.0)	1 (3.7)	—	0.248
Aplastic anaemia	1 (1.2)	0 (0.0)	—	1.000
Solid tumour (metastatic)	1 (1.2)	0 (0.0)	—	1.000
Solid tumour (localized)	0 (0.0)	1 (3.7)	—	0.248

Abbreviations: CDC: chronic disseminated candidiasis; OR: Odds Ratio

Antifungal susceptibility testing methods.

Antifungal susceptibility testing method was documented for 49/111 patients (44.1%). Among patients with a documented method, testing was performed using Sensititre YeastOne (15/111, 13.5%), Vitek 2 (12/111, 10.8%), EUCAST broth microdilution (9/111, 8.1%), CLSI broth microdilution (6/111, 5.4%), or E-test (3/111, 2.7%), reflecting the heterogeneity of routine mycology laboratory practice across the participating centres.

Only one *Candida glabrata* isolate was identified in the cohort. Susceptibility testing was performed by EUCAST broth microdilution, and the isolate was reported as fluconazole resistant (R), with an MIC >16 mg/L according to the EUCAST clinical breakpoint. No *C. glabrata* isolate was reported as susceptible, increased exposure (I).

Supplementary Table S4. Combination (overlapping) antifungal therapy for more than 3 days during treatment courses in 22 patients with CDC accounting for 43 overlapping episodes

Antifungal combination	Number of overlapping episodes	Median number of overlapping days
Liposomal amphotericin B + Isavuconazole	5	17
Liposomal amphotericin B + Voriconazole	4	12
Anidulafungin + Voriconazole	4	6
Liposomal amphotericin B + Flucytosine	4	21
Liposomal amphotericin B + Caspofungin	3	35
Liposomal amphotericin B + Anidulafungin	3	21
Flucytosine + Micafungin	2	13
Flucytosine + Voriconazole	2	12
Liposomal amphotericin B + Fluconazole	2	57
Anidulafungin + Fluconazole	1	15
Anidulafungin + Flucytosine	1	11
Anidulafungin + Isavuconazole	1	13
Anidulafungin + Posaconazole	1	8
Caspofungin + Fluconazole	1	107
Caspofungin + Posaconazole	1	5
Caspofungin + Voriconazole	1	21
Micafungin + Voriconazole	1	7
Fluconazole + Flucytosine	1	9
Fluconazole + Voriconazole	1	31
Isavuconazole + Micafungin	1	19
Isavuconazole + Rezafungin	1	109
Amphotericin B deoxycholate + Anidulafungin	1	41
Liposomal amphotericin B + Micafungin	1	18

Note: Parallel therapy was defined as concomitant administration of two antifungal agents with an overlap of more than 3 days within the same patient.

Combination antifungal therapy, defined as an overlap of more than 3 days between two antifungal agents within the same patient, was observed in 22 patients, accounting for 43 overlapping treatment episodes. In 16 patients, these concomitant therapies occurred during first line treatment. The most frequent combinations were liposomal amphotericin B with isavuconazole (n=5), liposomal amphotericin B with voriconazole (n=4), anidulafungin with voriconazole (n=4), and liposomal amphotericin B with flucytosine (n=4). Additional combinations included liposomal amphotericin B with caspofungin (n=3) and liposomal amphotericin B with anidulafungin (n=3). Other combinations occurred less frequently (≤2 episodes each) and involved echinocandins, azoles, amphotericin B formulations, or flucytosine.

Supplementary Table S5. Empirical antifungal therapy in nine patients with chronic disseminated candidiasis

**Variable**	**n/N**
First-line empirical therapy	9/111
Caspofungin	4/9
Liposomal amphotericin B	2/9
Voriconazole	2/9
Micafungin	1/9
Days; median, (range)	5 (1–19)
Second-line empirical therapy	8/9
Liposomal amphotericin B	4/8
Caspofungin	3/8
Posaconazole	1/8
Days; median, (range)	9 (1–15)

Empirical therapy was defined as any non-targeted antifungal therapy (i.e. not directed against CDC) administered before day 0.

Supplementary Table S6a. Cox proportional hazards model for overall survival (replication of Boussen et al.) [1]

Variable	HR	95% CI	p-value
Azole as first-line treatment	0.88	0.36–2.218	0.782
Adjunctive corticosteroids	0.89	0.33–2.38	0.816
Age at diagnosis	1.01	0.99–1.03	0.230
Neutropenia within 30 days prior CDC diagnosis	1.02	0.42–2.52	0.959
Other than “de novo” diagnosis of haemato-oncological disease at CDC diagnosis: remission, relapse, progression	1.65	0.84–3.25	0.150

Reference: amphotericin (all formulations) as first-line treatment

Abbreviations: CDC: chronic disseminated candidiasis; CI: confidence interval; HR: hazard ratio

Supplementary Table S6b. Cox proportional hazards model for overall survival including echinocandin as first-line treatment

Variable	HR	95% CI	p-value
Azole as first-line treatment	0.88	0.33–2.33	0.794
Echinocandin as first-line treatment	1.00	0.47–2.13	0.995
Adjunctive corticosteroids	0.89	0.33–2.40	0.817
Age at diagnosis	1.01	0.99–1.03	0.231
Neutropenia within 30 days prior CDC diagnosis	1.02	0.41–2.57	0.959
Other than “de novo” diagnosis of haemato-oncological disease at CDC diagnosis: remission, relapse, progression	1.65	0.83–3.25	0.151

Reference: amphotericin (all formulations) as first-line treatment

Abbreviations: CDC: chronic disseminated candidiasis; CI: confidence interval; HR: hazard ratio;.

Supplementary Table S6c (sensitivity analysis). Cox proportional hazards model for overall survival (replication of Boussen et al.) — proven/probable cohort (n=85)

Variable	HR	95% CI	p-value
Azole as first-line treatment	0.91	0.37–2.22	0.837
Adjunctive corticosteroids	0.84	0.31–2.25	0.728
Age at diagnosis	1.01	0.99–1.04	0.175
Neutropenia within 30 days prior CDC diagnosis	0.99	0.41–2.38	0.972
Other than “de novo” diagnosis of haemato-oncological disease at CDC diagnosis	1.67	0.85–3.30	0.140

Cox proportional hazards model for overall survival (replication of Boussen et al.), restricted to the sensitivity cohort of patients with proven or probable chronic disseminated candidiasis (n=85). Azole as first-line treatment, adjunctive corticosteroids, age at diagnosis, neutropenia within 30 days prior to diagnosis, and non-“de novo” haemato-oncological disease status were not significantly associated with overall survival, consistent with the primary cohort analysis.

Abbreviations: CDC: chronic disseminated candidiasis; CI: confidence interval; HR: hazard ratio. Reference: amphotericin B (all formulations) as first-line treatment.

Supplementary Table S6d (sensitivity analysis). Cox proportional hazards model for overall survival including echinocandin as first-line treatment — proven/probable cohort (n=85)

Variable	HR	95% CI	p-value
Azole as first-line treatment	0.99	0.33–2.93	0.964
Echinocandin as first-line treatment	0.89	0.39–2.02	0.779
Adjunctive corticosteroids	0.92	0.34–2.51	0.866
Age at diagnosis	1.01	0.98–1.03	0.637
Neutropenia within 30 days prior CDC diagnosis	1.16	0.35–3.89	0.805
Other than “de novo” diagnosis of haemato-oncological disease at CDC diagnosis	1.41	0.67–2.99	0.371

Neither echinocandin nor azole as first-line treatment was significantly associated with overall survival compared with amphotericin B, consistent with the corresponding analysis in the full cohort.

Reference: amphotericin B (all formulations) as first-line treatment.

Abbreviations: HR: hazard ratio; CDC: chronic disseminated candidiasis; CI: confidence interval.

Supplementary Table S6e (sensitivity analysis). Cox proportional hazards model for overall survival (replication of Boussen et al.) — known *Candida* species cohort (n=72)

Variable	HR	95% CI	p-value
Azole as first-line treatment	0.22	0.05–1.05	0.058
Adjunctive corticosteroids	0.70	0.20–2.42	0.575
Age at diagnosis	1.02	0.99–1.05	0.127
Neutropenia within 30 days prior CDC diagnosis	0.32	0.10–1.06	0.062
Other than “de novo” diagnosis of haemato-oncological disease at CDC diagnosis	3.63	1.47–8.94	0.005

Azole as first-line treatment, corticosteroids, age, and neutropenia were not significantly associated with overall survival; unlike the primary cohort, non-“de novo” haemato-oncological disease status reached nominal significance in this subgroup.

Reference: amphotericin B (all formulations) as first-line treatment.

Note: unlike the primary cohort and the proven/probable sensitivity cohort, this subgroup shows a nominally significant association between non-“de novo” haematological disease status and overall survival (HR 3.63, 95% CI 1.47–8.94, p=0.005), which was not significant in the primary cohort.

Abbreviations: CDC: chronic disseminated candidiasis; CI: confidence interval; HR: hazard ratio.

Supplementary Table S6f (sensitivity analysis). Cox proportional hazards model for overall survival including echinocandin as first-line treatment — known-*Candida* species cohort (n=72)

Variable	HR	95% CI	p-value
Echinocandin as first-line treatment	1.04	0.41–2.60	0.940
Azole as first-line treatment	0.22	0.04–1.18	0.077
Adjunctive corticosteroids	0.69	0.19–2.48	0.573
Age at diagnosis	1.02	0.99–1.05	0.128
Neutropenia within 30 days prior CDC diagnosis	0.32	0.10–1.06	0.061
Other than “de novo” diagnosis of haemato-oncological disease at CDC diagnosis	3.63	1.47–8.95	0.005

Abbreviations: HR: hazard ratio; CI: confidence interval. Reference: amphotericin B (all formulations) as first-line treatment.

Supplementary Table S6g (sensitivity analysis). Cox proportional hazards model for overall survival (replication of Boussen et al.) — cohort without combination therapy (n=89)

Variable	HR	95% CI	p-value
Azole as first-line treatment	1.171	0.451–3.039	0.746
Adjunctive corticosteroids	1.158	0.429–3.125	0.773
Age at diagnosis	1.015	0.992–1.039	0.197
Neutropenia within 30 days prior CDC diagnosis	1.345	0.448–4.036	0.597
Other than "de novo" diagnosis of haemato-oncological disease at CDC diagnosis: remission, relapse, progression	1.615	0.769–3.393	0.205

Reference: amphotericin (all formulations) as first-line treatment

Cohort restricted to the 89/111 patients (80.2%) who did not receive combination (overlapping) antifungal therapy for more than 3 days.

Abbreviations: CDC: chronic disseminated candidiasis; CI: confidence interval; HR: hazard ratio

Supplementary Table S6h (sensitivity analysis). Cox proportional hazards model for overall survival including echinocandin as first-line treatment — cohort without combination therapy (n=89)

Variable	HR	95% CI	p-value
Echinocandin as first-line treatment	0.681	0.293–1.583	0.372
Azole as first-line treatment	0.981	0.359–2.682	0.971
Adjunctive corticosteroids	1.241	0.451–3.417	0.676
Age at diagnosis	1.016	0.993–1.041	0.172
Neutropenia within 30 days prior CDC diagnosis	1.523	0.501–4.626	0.458
Other than "de novo" diagnosis of haemato-oncological disease at CDC diagnosis: remission, relapse, progression	1.595	0.754–3.372	0.222

Reference: amphotericin (all formulations) as first-line treatment

Abbreviations: CDC: chronic disseminated candidiasis; CI: confidence interval; HR: hazard ratio

Supplementary Table S7a. Treatment response over time (n = 111).

**Treatment response, n (%)**	**Day 14**	**Day 28**	**Day 42**	**Day 84**	**Month 6**	**Month 9**	**Month 12**
Complete response	4 (3.6)	7 (6.3)	13 (11.7)	14 (12.6)	24 (21.6)	22 (19.8)	23 (20.7)
Partial response	23 (20.7)	27 (24.3)	31 (27.9)	33 (29.7)	23 (20.7)	8 (7.2)	6 (5.4)
Stable disease	41 (36.9)	35 (31.5)	32 (28.8)	17 (15.3)	6 (5.4)	7 (6.3)	4 (3.6)
Progressive disease	27 (24.3)	20 (18.0)	8 (7.2)	8 (7.2)	3 (2.7)	1 (0.9)	3 (2.7)
Treatment completed; no formal response assessment	0 (0.0)	5 (4.5)	10 (9.0)	33 (29.7)	53 (47.7)	69 (62.2)	73 (65.8)
Unknown/ missing response assessment	16 (14.4)	17 (15.3)	17 (15.3)	6 (5.4)	2 (1.8)	4 (3.6)	2 (1.8)

Treatment response over time in the overall study cohort. This table provides the numerical values corresponding to the stacked bar chart shown in **Figure 3**. *Unknown/missing response assessment* denotes patients who remained under follow-up at the respective time point but for whom no formal response assessment was available. *Treatment completed; no formal response assessment* denotes patients who had discontinued antifungal therapy before the respective time point without a documented response assessment.

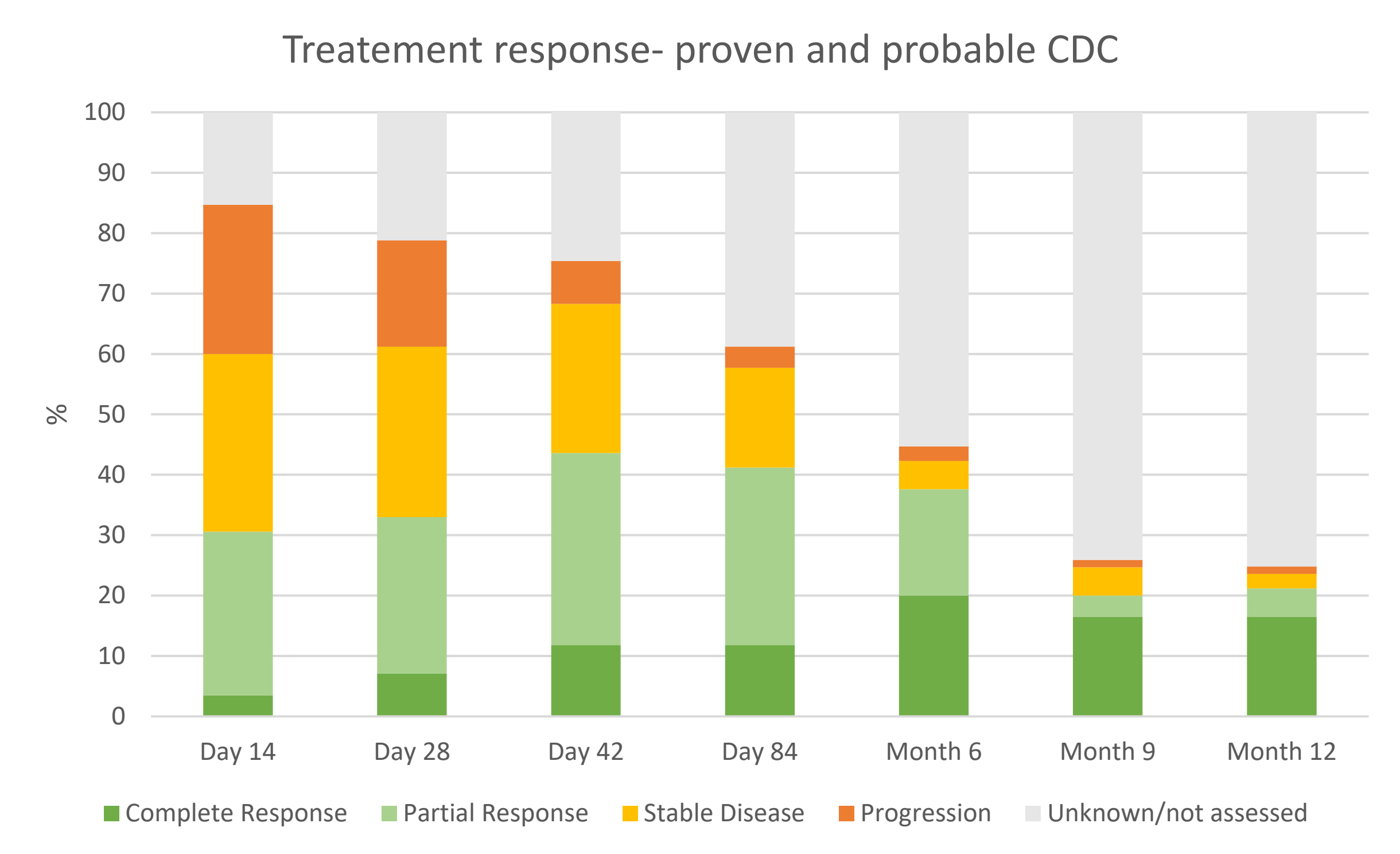

Supplementary Figure S2. Treatment response over time in patients with proven or probable chronic disseminated candidiasis (n=85; sensitivity analysis).

Stacked bar chart showing investigator-assessed treatment response at Day 14, Day 28, Day 42, Day 84, Month 6, Month 9, and Month 12 after excluding patients with possible chronic disseminated candidiasis. Treatment response was classified according to the predefined case report form as complete response, partial response, stable disease, or progression. Missing assessments at each time point include both patients with no documented response assessment despite ongoing follow-up and patients who had completed antifungal treatment before the respective time point without a formal response assessment. Percentages are calculated using the total sensitivity cohort (n=85) as the denominator, numerical values are presented below in Supplementary Table S7b.

Supplementary Table S7b. Treatment response over time in the sensitivity cohort restricted to proven and probable chronic disseminated candidiasis (n = 85).

Treatment response, n (%)	Day 14	Day 28	Day 42	Day 84	Month 6	Month 9	Month 12
Complete Response	3 (3.5)	6 (7.1)	10 (11.8)	10 (11.8)	17 (20.0)	14 (16.5)	14 (16.5)
Partial Response	23 (27.1)	22 (25.9)	27 (31.8)	25 (29.4)	15 (17.6)	3 (3.5)	4 (4.7)
Stable Disease	25 (29.4)	24 (28.2)	21 (24.7)	14 (16.5)	4 (4.7)	4 (4.7)	2 (2.4)
Progression	21 (24.7)	15 (17.6)	6 (7.1)	3 (3.5)	2 (2.4)	1 (1.2)	1 (1.2)
Missing	13 (15.3)	18 (21.2)	21 (24.7)	33 (38.8)	47 (55.3)	63 (74.1)	64 (75.3)

Supplementary Table S8a. Unweighted mixed-effects logistic regression model for treatment response day 84 through month 12.

Variable	OR	95% CI	p-value
Dominant antifungal class			
Amphotericin B	1.75	0.01–288.63	0.830
Echinocandin	3.18	0.02–472.89	0.651
Azole	3.32	0.03-443.02	0.630
Corticosteroids >50% of assessment window	0.67	0.05–9.21	0.764
Absolute neutrophil count <500 cells/µL	0.44	0.06–2.97	0.397

Outcome: binary treatment response (complete or partial response vs. stable disease or progression). Reference: no antifungal treatment. Immunosuppression as a time-dependent covariate could not be retained in the model due to instability, related to sparse events and limited variability over time. N = 75 patients, 148 observations

Supplementary Table S8b (sensitivity analysis). Unweighted mixed-effects logistic regression model for treatment response day 84 through month 12 — proven/probable cohort

Variable	OR	95% CI	p-value
**Dominant antifungal class (reference: no antifungal treatment)**			
Amphotericin B	—*	—	1.000
Echinocandin	—*	—	1.000
Azole	—*	—	1.000
Corticosteroids >50% of assessment window	0.35	0.04–3.46	0.368
Absolute neutrophil count <500 cells/µL	0.41	0.07–2.47	0.328

Unweighted mixed-effects logistic regression model for treatment response between day 84 and month 12, restricted to the proven/probable sensitivity cohort (n = 54 patients, 96 observations). Corticosteroid use for more than 50% of the assessment window and neutropenia were not significantly associated with treatment response. Estimates for first-line antifungal class could not be reliably obtained in this smaller subset owing to quasi-complete separation and are reported as non-estimable.

*The dominant-antifungal-class terms could not be reliably estimated in this restricted sensitivity sample: response was perfectly predicted by treatment class within some strata (quasi-complete separation),

yielding non-finite odds ratios and standard errors. Immunosuppression as a time-dependent covariate again could not be retained in the model due to instability. These results should be interpreted with caution and are best read as directionally consistent with, but statistically weaker/less stable than, the primary-cohort estimates.

Supplementary Table S8c (sensitivity analysis). Unweighted mixed-effects logistic regression model for treatment response day 84 through month 12 — known Candida species cohort

Variable	OR	95% CI	p-value
**Dominant antifungal class (reference: no antifungal treatment)**			
Amphotericin B	—*	—	1.000
Echinocandin	—*	—	1.000
Azole	—*	—	1.000
Corticosteroids >50% of assessment window	0.92	0.01–160.14	0.975
Absolute neutrophil count <500 cells/μL	0.42	0.02–10.61	0.599

N=48 patients, 96 observations. *The dominant-antifungal-class terms could not be reliably estimated owing to quasi-complete separation.

Supplementary Table S8d (sensitivity analysis). Unweighted mixed-effects logistic regression model for treatment response day 84 through month 12 — cohort without combination therapy

Variable	OR	95% CI	p-value
Dominant antifungal class			
Amphotericin B	1.446	0.008–256.912	0.889
Echinocandin	3.862	0.032–468.854	0.581
Azole	3.594	0.033–385.877	0.592
Corticosteroids >50% of assessment window	1.242	0.045–34.068	0.898
Absolute neutrophil count <500 cells/μL	0.920	0.077–11.052	0.948

N=59 patients, 118 observations Cohort restricted to patients who did not receive combination (overlapping) antifungal therapy for more than 3 days. Note the markedly wide confidence intervals, reflecting sparse data within this smaller subgroup; estimates should be interpreted with caution.

Abbreviations: CDC: chronic disseminated candidiasis; CI: confidence interval; OR: odds ratio

Supplementary Table S9a. IPTW-weighted marginal structural model adjusted for baseline covariates (BDG, ICU stay, unknown *Candida* species) for treatment response day 84 to month 12.

Variable	OR	95% CI	p-value
Dominant antifungal class			
Amphotericin B	Reference	–	–
Echinocandin	2.25	0.35–14.58	0.377
Azole	1.26	0.26–6.16	0.766
BDG positive at CDC diagnosis	0.21	0.02–2.16	0.176
ICU stay	0.16	0.01–2.23	0.162
Candida species unknown	0.74	0.16–3.49	0.692

N=26 patients, 40 observations (complete cases).

Reference: amphotericin (all formulations).

Abbreviations: BDG: beta-D-glucan; CDC: chronic disseminated candidiasis, ICU: intensive care unit.

Supplementary Table S9b (sensitivity analysis). IPTW-weighted marginal structural model adjusted for baseline covariates (BDG, ICU stay, unknown Candida species) for treatment response day 84 to month 12 — proven/probable cohort

Variable	OR	95% CI	p-value
Echinocandin	—*	—	<0.001
Azole	—*	—	<0.001
BDG positive at CDC diagnosis	—*	—	<0.001
ICU stay	—*	—	<0.001
Candida species unknown	0.16	0.01–1.80	0.124

N=17 patients, 24 observations. Unknown *Candida* species remained non-significantly associated with lower odds of response, consistent with the primary analysis. Estimates for treatment class, BDG positivity, and ICU stay were non-estimable in this reduced sample owing to sparse data.

Reference: amphotericin B (all formulations).

Given N=17, this model is substantially underpowered its point estimates should not be over-interpreted; Presenting this sensitivity result mainly to show that no qualitatively different conclusion emerges.

Abbreviations: BDG: beta-D-glucan; CDC: chronic disseminated candidiasis; ICU: intensive care unit

Supplementary Table S9c (sensitivity analysis). IPTW-weighted marginal structural model for treatment response day 84 to month 12 — known *Candida* species cohort

Variable	OR	95% CI	p-value
Echinocandin	6.45	0.78–53.05	0.077
Azole	2.81	0.56–14.21	0.186
BDG positive at CDC diagnosis	0.41	0.03–6.20	0.481
ICU stay	0.15	0.01–3.58	0.213

N=15 patients, 24 observations.

Reference: amphotericin B (all formulations).

None of the estimates reach statistical significance, though echinocandin and azole point in the direction of higher odds of response relative to amphotericin B (consistent with, but not significantly different from, the primary-cohort direction), and BDG positivity and ICU stay trend towards lower odds of response, consistent with the primary analysis.

Abbreviations: BDG: beta-D-glucan; CDC: chronic disseminated candidiasis; ICU: intensive care unit.

Supplementary Table S9d (sensitivity analysis). IPTW-weighted marginal structural model for treatment response day 84 to month 12 — cohort without combination therapy

Variable	OR	95% CI	p-value
Dominant antifungal class			
Amphotericin B	0.891	0.094–8.404	0.918
Echinocandin	1.941	0.231–16.325	0.535
Azole	1.482	0.169–13.030	0.718
Corticosteroids >50% of assessment window	0.607	0.095–3.871	0.591
Absolute neutrophil count <500 cells/µL	0.531	0.112–2.510	0.417

Cohort restricted to the 89/111 patients (80.2%) who did not receive combination (overlapping) antifungal therapy for more than 3 days. This model included 59 evaluable patients and 118 observations (repeated response assessments per patient across timepoints

Abbreviations: CDC: chronic disseminated candidiasis; CI: confidence interval; OR: odds ratio

Supplementary Table S10a (sensitivity analysis). Propensity score-weighted analysis of first to second-line sequential treatment strategies and treatment response at day 84 months — proven/probable cohort

Treatment switch strategy	N	Included in analysis	OR	95% CI	p-value
**Strategy A: Initial treatment switch from caspofungin or liposomal amphotericin B (n = 14 patients)**					
Caspofungin → fluconazole	6	—	Reference	—	—
LAMB → fluconazole	2	—	—*	—	*
Caspofungin → other azoles	3	—	—*	—	*
L-AMB → other azoles	3	—	—*	—	*
**Strategy B: Initial treatment switch from echinocandin or amphotericin B (drug classes) (n = 22 patients)**					
Echinocandin → fluconazole	9	—	Reference	—	—
Amphotericin B (all) → fluconazole	2	—	—†	—	<0.001†
Echinocandin → other azoles	5	—	0.90	0.06–14.09	0.938
Amphotericin B (all) → other azoles	6	—	1.46	0.09–23.15	0.778
**Strategy C: Collapsed model — Initial treatment switch from echinocandin or amphotericin B (n = 22 patients)**					
Echinocandin/amphotericin B → fluconazole	11	11	Reference	—	—
Echinocandin/amphotericin B → other azoles	11	11	0.96	0.09–10.02	0.969

In the collapsed comparison (Strategy C), switching to fluconazole versus another azole after initial echinocandin or amphotericin B therapy was not significantly associated with treatment response, consistent with the primary cohort. The more granular switch strategies (A and B) could not be reliably estimated in this reduced sample because of small subgroup sizes.

*Strategy A (n = 14 patients) could not be reliably fit as a weighted logistic model in this reduced sensitivity sample: the switch categories are strongly confounded with response (quasi-complete separation

†In Strategy B, the amphotericin B (all) → fluconazole group (n = 2) is likewise non-estimable because of complete separation with only 2 patients; unlike the primary-cohort analysis, this could not be resolved even qualitatively given the smaller sensitivity sample.

Supplementary Table S10b (sensitivity analysis). Propensity score-weighted analysis of first to second-line sequential treatment strategies and treatment response at day 84/3 months — known *Candida*-species

Treatment switch strategy	N	Included in analysis	OR	95% CI	p-value
**Strategy A: Initial treatment switch from caspofungin or liposomal amphotericin B (n = 13 patients)**					
Caspofungin → fluconazole	4	—	Reference	—	—
LAMB → fluconazole	3	—	—*	—	*
Caspofungin → other azoles	3	—	—*	—	*
L-AMB → other azoles	3	—	—*	—	*
**Strategy B: Initial treatment switch from echinocandin or amphotericin B (drug classes) (n = 22 patients)**					
Echinocandin → fluconazole	7	—	Reference	—	—
Amphotericin B (all) → fluconazole	4	—	—†	—	<0.001†
Echinocandin → other azoles	6	—	1.36	0.06–29.26	0.833
Amphotericin B (all) → other azoles	5	—	8.49	0.23–321.09	0.230
**Strategy C: Collapsed model — Initial treatment switch from echinocandin or amphotericin B (n = 22 patients)**					
Echinocandin/amphotericin B → fluconazole	11	11	Reference	—	—
Echinocandin/amphotericin B → other azoles	11	11	0.78	0.07–8.28	0.827

Outcome: binary treatment response (complete or partial response vs. stable disease or progression) at day 84/3 months, restricted to the known-species sensitivity cohort.

*Strategy A (n = 13 patients) could not be reliably fit in this reduced sensitivity sample: the model failed to converge, with extreme non-finite odds ratios reflecting quasi-complete separation; only raw switch counts are reported. This mirrors the pattern seen in the proven/probable sensitivity cohort.

†In Strategy B, the amphotericin B (all) → fluconazole group (n = 4) is non-estimable because of complete separation with this switch category; the remaining two categories in Strategy B (echinocandin/amphotericin B → other azoles) are estimable and both show non-significant associations, in the same direction of no clear benefit for either switch pattern seen in the other sensitivity analyses.

Supplementary Table S10c (sensitivity analysis). Propensity score-weighted analysis of first to second-line sequential treatment strategies and treatment response — cohort without combination therapy

Treatment switch strategy	N	Included in analysis	OR	95% CI	p-value
**Strategy A: Initial treatment switch from caspofungin or liposomal amphotericin B (n = 17 patients)**					
Caspofungin → fluconazole	7	—	Reference	—	—
LAMB → fluconazole	3	—	—*	—	*
Caspofungin → other azoles	4	—	1.04	0.04–30.4	0.981
L-AMB → other azoles	3	—	0.80	0.03–19.4	0.878
**Strategy B: Initial treatment switch from echinocandin or amphotericin B (drug classes) (n = 23 patients)**					
Echinocandin → fluconazole	10	—	Reference	—	—
Amphotericin B (all) → fluconazole	4	—	—†	—	<0.001†
Echinocandin → other azoles	5	—	2.45	0.19–30.8	0.466
Amphotericin B (all) → other azoles	4	—	1.19	0.05–31.7	0.911
**Strategy C: Collapsed model — Initial treatment switch from echinocandin or amphotericin B (n = 23 patients)**					
Echinocandin/amphotericin B → fluconazole	14	14	Reference	—	—
Echinocandin/amphotericin B → other azoles	9	9	1.75	0.18–17.4	0.615

Outcome: binary treatment response (complete or partial response vs. stable disease or progression) at day 84/3 months, restricted to the sensitivity cohort of patients without combination (overlapping) antifungal therapy for more than 3 days. In the collapsed comparison (Strategy C), switching to fluconazole versus another azole after initial echinocandin or amphotericin B therapy was not significantly associated with treatment response (OR 1.75, 95% CI 0.18–17.4, p=0.615), consistent with the primary cohort and other sensitivity analyses. The more granular switch strategies (A and B) could not be reliably estimated in this reduced sample because of small subgroup sizes.

*Strategy A (n = 17 patients) could not be reliably fit as a weighted logistic model in this reduced sensitivity sample: the liposomal amphotericin B → fluconazole category (n = 3) produced an extreme, non-finite odds ratio due to complete separation; only the raw switch count is reported for this category. The remaining two categories were estimable and showed no significant association with treatment response (caspofungin → other azoles: OR 1.04, 95% CI 0.04–30.4, p=0.981; liposomal amphotericin B → other azoles: OR 0.80, 95% CI 0.03–19.4, p=0.878).

†In Strategy B, the amphotericin B (all) → fluconazole group (n = 4) is likewise non-estimable because of complete separation; only the raw switch count is reported for this category. The remaining two

categories were estimable and showed no significant association with treatment response (echinocandin → other azoles: OR 2.45, 95% CI 0.19–30.8, p=0.466; amphotericin B (all) → other azoles: OR 1.19, 95% CI 0.05–31.7, p=0.911).

Supplementary Table S11a. Univariate IPTW-weighted models for treatment response (day 84 through month 12) — baseline covariates.

Variable	OR	95% CI	p-value (BH-adj.)
No treatment (reference)	Reference	—	—
Amphotericin B	0.92	0.11–7.39	0.976
Echinocandin	1.53	0.19–12.23	0.940
Azole	1.56	0.19–13.0	0.940
Age at diagnosis (per 1-year increase)	0.99	0.96–1.03	0.976
Male sex	0.57	0.18–1.78	0.574
Haematological/oncological disease (any)	11.65	0.62–218.2	0.330
De novo disease	0.97	0.34–2.78	0.946
Duration since haematological/ oncological diagnosis (per month)	1.00	0.97–1.04	0.913
Acute myeloid leukaemia	0.72	0.24–2.12	0.737
Acute lymphoblastic leukaemia	1.40	0.42–4.65	0.753
Lymphoma	1.11	0.16–7.53	0.946
Allogeneic HCT	1.24	0.20–7.48	0.913
Autologous HCT	0.46	0.09–2.50	0.574
GvHD within 90 days prior to CDC	0.00	0.00–0.00	—†
Neutropenia within 30 days prior	3.35	0.53–21.08	0.446
Neutropenia at diagnosis (ANC <500 cells/µL)	0.41	0.12–1.41	0.446
Antifungal prophylaxis within 14 days prior	1.85	0.49–7.04	0.574
Antibiotics within 14 days prior	0.51	0.15–1.74	0.556
Corticosteroids ≥0.3 mg/kg/day ≥3 weeks (within 60 days)	4.55	1.01–20.49	0.243

Non-chemotherapy immunosuppressive drugs (within 3 months)	2.19	0.20–24.37	0.737
ICU stay	0.07	0.02–0.25	0.001
Candida species unknown	0.54	0.17–1.75	0.560
C. albicans	0.48	0.15–1.58	0.477
C. tropicalis	4.19	0.91–19.21	0.278
Blood culture positive within prior 14 days of CDC or at diagnosis	2.52	0.67–9.48	0.446
Beta-D-glucan positive	0.00	0.00–0.00	—†
GGT (elevated)	0.24	0.03–2.12	0.446
Alkaline phosphatase (elevated)	0.85	0.18–3.94	0.913

BH-adjusted p-values calculated across all listed covariates using Benjamini-Hochberg procedure. Reference category for antifungal class: no treatment (primary model) or amphotericin (baseline covariate model).

† Complete separation: the variable perfectly predicts the outcome in one or more cells; OR and CI are unreliable and not reported. These variables (GvHD within 90 days, BDG positivity) were excluded from the multivariable model.

Abbreviations: ANC: absolute neutrophil count; BH-adj.: Benjamini-Hochberg adjusted; CDC: chronic disseminated candidiasis; CI: confidence interval; GGT: gamma-glutamyl transferase; GvHD: graft-versus-host disease; HCT: haematopoietic cell transplantation; ICU: intensive care unit; OR: odds ratio.

Supplementary Table S11b (sensitivity analysis). Univariate IPTW-weighted models for treatment response (day 84 through month 12) — baseline covariates, proven/probable cohort

Variable	OR	95% CI	p-value (BH-adj.)
Age at diagnosis (per 1-year increase)	0.99	0.95–1.04	0.906
Male sex	0.48	0.12–1.87	0.843
Haematological/oncological disease (any)	—‡	—	—
De novo disease	1.42	0.38–5.25	0.887
Duration since haematological/oncological diagnosis (per month)	0.99	0.96–1.03	0.906
Acute myeloid leukaemia	0.30	0.06–1.47	0.585

Variable	OR	95% CI	p-value (BH-adj.)
Acute lymphoblastic leukaemia	1.18	0.23–6.18	0.906
Lymphoma	2.64	0.15–46.79	0.887
Allogeneic HCT	0.55	0.09–3.51	0.887
Autologous HCT	1.35	0.11–16.20	0.906
GvHD within 90 days prior to CDC	0.00	0.00–0.00	—†
Neutropenia within 30 days prior	2.88	0.31–26.47	0.887
Neutropenia at diagnosis (ANC <500 cells/µL)	0.24	0.05–1.10	0.443
Antifungal prophylaxis within 14 days prior	1.87	0.43–8.09	0.887
Antibiotics within 14 days prior	0.64	0.14–2.99	0.887
Corticosteroids ≥0.3 mg/kg/day ≥3 weeks (within 60 days)	3.66	0.61–22.02	0.585
Non-chemotherapy immunosuppressive drugs (within 3 months)	0.39	0.03–4.75	0.887
ICU stay	0.17	0.04–0.72	0.163
Candida species unknown	0.56	0.15–2.07	0.887
C. albicans	1.22	0.28–5.36	0.906
C. tropicalis	1.63	0.31–8.45	0.887
Blood culture positive within prior 14 days of CDC or at diagnosis	1.04	0.18–5.90	0.963
Beta-D-glucan positive	—‡	—	—
GGT (elevated)	0.60	0.07–5.11	0.894
Alkaline phosphatase (elevated)	0.28	0.05–1.57	0.585

Univariate IPTW-weighted logistic regression models for treatment response between day 84 and month 12, one baseline covariate at a time, restricted to the proven/probable sensitivity cohort. p-values are Benjamini–Hochberg adjusted across all covariates tested in this sensitivity run. As in the primary-cohort Table S8, ICU stay and several disease/treatment covariates show non-significant associations after adjustment for multiplicity; unlike the primary cohort, ICU stay no longer reaches nominal significance in this smaller subset, given the wide, overlapping confidence intervals, these differences most plausibly reflect sampling variability in a reduced sample rather than a genuine effect-modification by CDC certainty category.

The univariate test of antifungal class itself (No treatment/Amphotericin B/Echinocandin/Azole, reported as the first four rows of the primary-cohort Table S11a) is not separately reported here: in this sensitivity run the ‘covariate’ term for antifungal class was collinear with the model’s existing exposure term,

producing uninterpretable estimates; the corresponding antifungal-class associations are instead reported in the Tables above.

†Complete separation: GvHD within 90 days before CDC perfectly predicts the outcome in this subgroup, as in the primary cohort; OR and CI are not reliable and are shown as 0.00 for reference only, consistent with the primary-cohort table.

‡Could not be estimated in this subgroup (model fitting failed) owing to near-complete lack of variation in haematological/oncological disease (present in almost all patients) and beta-D-glucan positivity within this smaller sensitivity cohort.

Abbreviations: ANC: absolute neutrophil count; BH-adj.: Benjamini–Hochberg adjusted; CDC: chronic disseminated candidiasis; CI: confidence interval; GGT: gamma-glutamyl transferase; GvHD: graft-versus-host disease; HCT: haematopoietic cell transplantation; ICU: intensive care unit; OR: odds ratio.

Supplementary Table S11c (sensitivity analysis). Univariate IPTW-weighted models for treatment response (day 84 through month 12) — baseline covariates, known *Candida* species cohort

Variable	OR	95% CI	p-value (BH-adj.)
Age at diagnosis (per 1-year increase)	0.99	0.95–1.02	0.666
Male sex	0.87	0.23–3.30	0.904
Haematological/oncological disease (any)	—‡	—	—
De novo disease	1.43	0.40–5.20	0.766
Duration since hematological/oncological diagnosis (per month)	1.04	0.93–1.16	0.750
Acute myeloid leukaemia	0.86	0.19–3.93	0.904
Acute lymphoblastic leukaemia	2.38	0.51–11.10	0.525
Lymphoma	1.04	0.08–13.22	0.977
Allogeneic HCT	0.82	0.13–5.17	0.904
Autologous HCT	0.38	0.04–3.92	0.631
GvHD within 90 days prior to CDC	0.00	0.00–0.00	—†
Neutropenia within 30 days prior	2.14	0.08–56.06	0.816
Neutropenia at diagnosis (ANC <500 cells/µL)	0.36	0.08–1.61	0.406
Antifungal prophylaxis within 14 days prior	2.36	0.36–15.61	0.599
Antibiotics within 14 days prior	0.46	0.10–2.12	0.541
Corticosteroids ≥0.3 mg/kg/day ≥3 weeks (within 60 days)	2.90	0.39–21.47	0.540

Variable	OR	95% CI	p-value (BH-adj.)
Non-chemotherapy immunosuppressive drugs (within 3 months)	1.08	0.06–18.34	0.977
ICU stay	0.04	0.01–0.23	0.002
C. albicans	0.25	0.06–1.07	0.172
C. tropicalis	4.21	0.83–21.27	0.205
Blood culture positive within prior 14 days of CDC or at diagnosis	2.64	0.59–11.84	0.431
Beta-D-glucan positive	—‡	—	—
GGT (elevated)	—‡	—	—
Alkaline phosphatase (elevated)	0.82	0.13–5.23	0.904

Univariate IPTW-weighted logistic regression models for treatment response between day 84 and month 12, one baseline covariate at a time, restricted to the known-species sensitivity cohort. p-values are Benjamini–Hochberg adjusted across all covariates tested in this sensitivity run. The '*Candida* species unknown' covariate is not applicable in this cohort and is omitted.

The univariate test of antifungal class itself (as in the primary-cohort Table S11a) is not separately reported here for the same reason given for the other sensitivity cohorts: collinearity with the model's existing exposure term produced uninterpretable estimates in this run.

†Complete separation: GvHD within 90 days before CDC perfectly predicts the outcome in this subgroup, as in the primary and proven/probable-cohort tables.

‡Could not be estimated in this subgroup (model fitting failed): hematological/oncological disease (present in nearly all patients), beta-D-glucan positivity, and — unlike the other two cohorts — GGT (elevated) also failed to converge here, likely reflecting sparse events in this smaller, more homogeneous subgroup.

Abbreviations: ANC: absolute neutrophil count; BH-adj.: Benjamini–Hochberg adjusted; CDC: chronic disseminated candidiasis; CI: confidence interval; GGT: gamma-glutamyl transferase; GvHD: graft-versus-host disease; HCT: hematopoietic cell transplantation; ICU: intensive care unit; OR: odds ratio. Each row is a separate univariate IPTW-weighted model; confidence intervals are not multiplicity-adjusted, only the p-values are.

Supplementary Table S12. Comparison of the present cohort with previously published chronic disseminated candidiasis / hepatosplenic candidiasis series, among the largest reported to date.

Characteristic	This study 2025	Pagano 2002 [2]	Della Pepa 2016 [3]	Chen 2019 [4]	Boussen 2022 [1]	Rammaert 2022 [5]	Cohen 2023 [6]	Chen 2025 [7]
**Design & setting**	Multinational registry, 19 countries	4 centres, Italy	Multicentre registry, Italy (35 centres surveyed; 7 contributed cases)	Single centre, Taiwan	Single centre, France	Multicentre prospective pilot, France (23 centres)	2 centres, Israel	Single centre, China
**Study period**	2007–2025	1990–2000	2003–2013	2008–2013	2008–2020	2013–2017	2005–2020	2014–2023
**Patients, n**	111	28 (29 episodes)	20	61	60	44 evaluable (52 enrolled)	35	49
**Age, median, years**	49 (IQR 36-59)	42 (range 17–67)	50 (range 19–62)	51 (range 18–83)	mean 44·5 (SD 17·7)	mean 46·9 (SD 16·3)	29 (IQR 15–45)	23(range 2–61)
**Proven / probable / possible CDC (EORTC), %**	26·1 / 50·5 / 23·4	64·3 / 28·6 / 7·1	35 / 65 / 0	4·9 / 21·3 / 73·8	18·3 / 3·3 / 78·3	0 / 45·5 / 54·5	Not reported	10·2 / 6·1 / 83·7
**Predominant *Candida* species**	*C. albicans* 32·4%, *C. tropicalis* 24·3%	*C. albicans* predominant (not systematically speciated)	*C. albicans* 45%, *C. krusei* 25%	*C. tropicalis* predominant among bloodstream isolates (8/13)	*Candida* colonization*:* *C. albicans* 79·2%, *C. glabrata* 10·4%	Not reported	*C. tropicalis* 50%	Not reported *(C. tropicalis* 6.1%, *C. albicans* 2.0% among previous candidemia)
**Previous candidemia, %**	29·7	32 (median latency 20 d, range 13–32)	Not reported	21·3	36·7	16	<33	16·3
**Most frequent first-line antifungal**	Echinocandin (58·6%)	Amphotericin B deoxycholate (68%)	Liposomal amphotericin B (45%)	Fluconazole (57·3%)	Azole (61·7%)	Not reported	Not reported	Azole (67·3%)

**Adjunctive corticosteroids, %**	4·5 (IRIS)	Not reported	Not reported	Not reported	45	Overall, not reported; 53·8 (7/13) among patients with normalized PET-CT at M3	33	4
**Median antifungal treatment duration**	107 d (IQR 49–182)	Not reported (oral fluconazole maintenance ≥3 months after IV induction)	30 d (range 10–90)	96 d (range 7–796)	Mean 7·4 mo (±6·0)	82·9% treated >M3; in patients with normalized PET-CT at M3: 60 d (IQR 50-63)	Mean total disease duration 178 days†	123 d (range 11–700)
**All-cause mortality, %**	33·3	14·3 (30-day)	60 (at 90 days)	19·7 (90-day)	36·7	25·0	Not reported	16·3
**CDC-attributable mortality, %**	4·5	3·6	15	Not reported	0	0	Not reported	0

Data are % unless otherwise indicated. †Treatment duration not explicitly reported.

Abbreviations: EORTC, European Organization for Research and Treatment of Cancer; IQR, interquartile range; IRIS, immune reconstitution inflammatory syndrome; M3: 3 months; CDC-attributable mortality denotes death directly attributed to CDC.

References

1. Boussen I, Lisan Q, Raffoux E, et al. Hepatosplenic Candidiasis in Patients With Hematological Malignancies: A 13-Year Retrospective Cohort Study. *Open Forum Infect Dis* 2022; **9**(4): ofac088.
2. Pagano L, Mele L, Fianchi L, et al. Chronic disseminated candidiasis in patients with hematologic malignancies. Clinical features and outcome of 29 episodes. *Haematologica* 2002; **87**(5): 535-41.
3. Della Pepa R, Picardi M, Sorà F, et al. Successful management of chronic disseminated candidiasis in hematologic patients treated with high-dose liposomal amphotericin B: a retrospective study of the SEIFEM registry. *Support Care Cancer* 2016; **24**(9): 3839-45.
4. Chen CY, Cheng A, Tien FM, et al. Chronic disseminated candidiasis manifesting as hepatosplenic abscesses among patients with hematological malignancies. *BMC Infect Dis* 2019; **19**(1): 635.
5. Rammaert B, Maunoury C, Rabeony T, et al. Does (18)F-FDG PET/CT add value to conventional imaging in clinical assessment of chronic disseminated candidiasis? *Front Med (Lausanne)* 2022; **9**: 1026067.
6. Cohen N, Orenbuch-Harroch E, Olshtain-Pops K, Lachish T, Korem M. Epidemiology, Clinical Characteristics and Risk Factors for Severity of Chronic Disseminated Candidiasis in Jerusalem, Israel. *Mycopathologia* 2023; **188**(6): 873-83.
7. Chen Z, Zhen S, Sun J, et al. Clinical characteristics and treatment response of chronic disseminated candidiasis in patients with hematological disorders. *Sci Rep* 2025; **15**(1): 12868.
